# Polymorphic transitions of diborane at sub- and near-megabar pressures

**DOI:** 10.1038/srep13929

**Published:** 2015-09-10

**Authors:** Amin Torabi, Chitra Murli, Yang Song, Viktor N. Staroverov

**Affiliations:** 1 Department of Chemistry, The University of Western Ontario, London, Ontario N6A 5B7, Canada; 2 High Pressure & Synchrotron Radiation Physics Division, Bhabha Atomic Research Centre, Mumbai, 400085, India

## Abstract

Recent theoretical investigations of high-pressure structures of diborane have yielded many intriguing predictions which have so far remained untested due to challenges of acquiring experimental data at extreme pressures. Here we report new pressure-induced polymorphic transformations of crystalline diborane observed between 36 and 88 GPa by *in situ* Raman spectroscopy and interpreted using electronic structure calculations. Two previously unknown phase transitions are identified near 42 and 57 GPa, as evidenced by significant changes in the Raman profiles. The corresponding new phases, labeled IV and V, consist of B_2_H_6_ molecules and have triclinic unit cells (***P***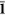), as deduced through evolutionary structure search and comparison of experimental and simulated Raman spectra. Density-functional calculations suggest that, at pressures above 110 GPa, phase V will form new molecular structures consisting of one-dimensional (BH_3_)_*n*_ chains and will become metallic near 138 GPa. Our findings make a significant contribution to the elucidation of the structures and properties of diborane in the near-megabar pressure region.

Chemical hydrides under extreme pressures have become the subject of explosive interest in recent years. Rich in elemental hydrogen, these materials offer attractive possibilities for high-density energy storage^1^. Being “chemically pre-compressed”^2^, they also provide a practical route to achieving pressure-induced hydrogen metallization, a long-standing challenge of high-pressure physics. In this context, experimental and theoretical studies of compressed silane (SiH_4_) and other hydrides have already yielded notable discoveries^3^,^4^,^5^,^6^,^7^,^8^,^9^.

Diborane (B_2_H_6_), a peculiar electron-deficient molecule with bridging hydrogen atoms, is another chemical hydride that attracts much attention in high-pressure research^10^,^11^,^12^,^13^,^14^,^15^,^16^. At ambient pressure and below 60 K, solid diborane crystallizes as an orthorhombic structure referred to as the α-phase, while annealing to above 90 K results in the formation of the β-phase (space group *P*2_1_/*n*, *a* = 4.40 Å, *b* = 5.72 Å, *c* = 6.50 Å, γ = 105.1°)^17^,^18^. Song and co-workers^11^,^12^ were the first to report intriguing pressure-induced phase transitions in diborane. Specifically, they found that diborane progresses through three crystal structure phases (labeled I, II, and III) in the pressure region between 3.5 GPa (the liquid−solid boundary at room temperature) and 24 GPa. Using density-functional calculations, Torabi *et al.*^15^ identified phase I with the *β*-diborane structure^17^ and found that phases II (formed at 6 GPa) and III (formed at 14 GPa) both have monoclinic *P*2_1_/*c* structures differing by molecular orientations. Abe and Ashcroft^13^ used density-functional theory to show that diborane is thermodynamically unstable with respect to decomposition into B and H between 40 and 350 GPa, but also predicted that the metastable B_2_H_6_ structures should become metallic around 160 GPa. Yao and Hoffmann^14^ demonstrated the thermodynamic possibility of oligo- and polymerization of B_2_H_6_ units above 4 GPa. Torabi *et al.*^15^ arrived at the same conclusions, but showed that phases II and III were still molecular crystals of B_2_H_6_. More recently, Hu *et al.*^16^ suggested that B_2_H_6_ should decompose into BH and H_2_ at 153 GPa and that the most stable structure of BH should become metallic at 168 GPa.

Despite these theoretical predictions, no direct evidence of non-dimer-based phases of boron hydride has been obtained to date due to experimental challenges in both sample confinement and *in situ* structural characterization under extreme pressures. In particular, it is very difficult to unambiguously locate atoms of light elements such as boron and hydrogen by X-ray diffraction. As a result, the structure of diborane beyond 50 GPa remains controversial. Here we report the first experimental evidence of new polymorphs of diborane using an alternative yet highly sensitive spectroscopic probe in a wide pressure regime approaching one megabar, which is unprecedented for this class of compounds. Our interpretation of the experimental data using electronic structure calculations suggests that B_2_H_6_ molecular units persist beyond 100 GPa, but transform into one-dimensional (BH_3_)_*n*_ chains near 110 GPa, and that the latter polymorph becomes metallic at even higher pressures. These findings shed light on the previously unknown high-pressure structures of diborane and take us one step closer to solving the problem of hydrogen metallization.

## Results

**Figure 1:**
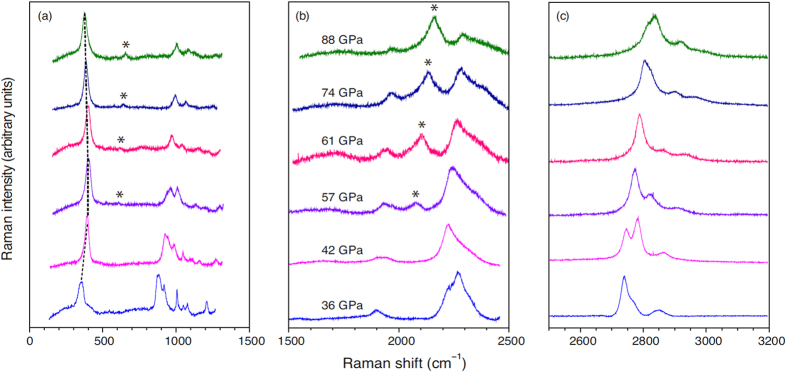
Experimental Raman spectra of compressed diborane. Raman spectra recorded in three frequency regions at selected pressures between 36 and 88 GPa. The dashed lines in panel (**a**) trace the soft behavior of the lattice mode. The asterisks label the new bands emerging near 57 GPa.

After gaseous diborane was cryogenically loaded into a diamond anvil cell, we succeeded in compressing the sample stepwise to about 90 GPa. *In situ* Raman spectroscopy was used to monitor the structural changes as a function of pressure (see Methods). The Raman spectra of diborane recorded upon compression at selected pressures between 36 and 88 GPa are presented in Fig. 1. At the starting pressure of 36 GPa, we deal with the known phase III (*P*2_1_/*c*)^15^, as evidenced by the fact that the experimental Raman profile of the sample at 36 GPa is best reproduced by the simulated Raman spectrum of phase III for the same pressure (see Supplementary Section S3). Upon compression of phase III to 42 GPa, most Raman modes below 2000 cm^−1^ blue-shift and broaden; other significant changes include a red shift and a profile change of the symmetric ring-stretching mode near 2250 cm^−1^, and the splitting of the terminal symmetric BH stretching mode near 2750 cm^−1^ (Fig. 1). These observations suggest a polymorphic transition at 42 GPa to a new phase, which we label phase IV.

Upon compression of phase IV to 57 GPa, another set of prominent changes was observed. The first of these is the appearance of two new Raman modes near 600 and 2100 cm^−1^, which are marked with asterisks in Fig. 1. With increasing pressure, the latter mode evolves into a very intense band. More interestingly, the doublet of the terminal BH symmetric stretching mode near 2750 cm^−1^ undergoes an intensity reversal between the two components at 57 GPa. Finally, the lowest-frequency lattice mode exhibits a soft turning behavior near 57 GPa, which is usually indicative of a phase transition. These observations suggest that another phase is formed near 57 GPa, which we label phase V. Given the similarity in Raman profiles recorded between 57 and 88 GPa, we conclude that no other phase transition occurs in that pressure range.

**Figure 2:**
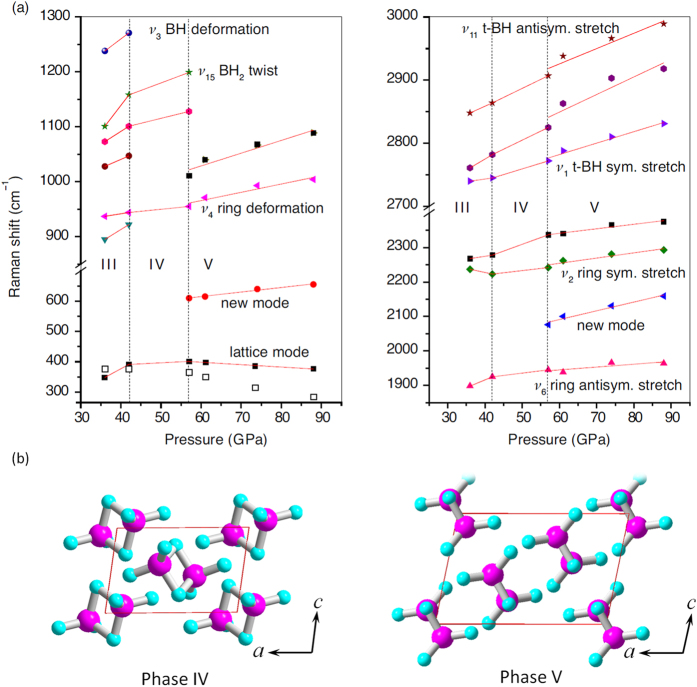
Experimental Raman shifts of compressed diborane as functions of pressure. (**a**) The calculated frequencies of the lattice mode are shown in the left panel as open squares. (**b**) Structures assigned to phases IV and V.

Further experimental evidence of the formation of phases IV and V is provided by the pressure-dependence plots for Raman shifts (Fig. 2). These plots reveal two phase boundaries in the 36–88 GPa range: one at 42 GPa and another at 57 GPa, denoted by the vertical dashed lines. Note that in the previous experimental studies^11^ of compressed diborane using infrared (IR) spectroscopy at pressures up to 50 GPa, a phase transition near 42 GPa was not observed. This is not inconsistent with the present findings because IR absorption bands at pressures above 30 GPa were so broad that IR data alone could neither indicate nor rule out a phase transition.

To assign possible crystal structures to phases IV and V, we proceeded as follows. First, we ran the evolutionary structure search algorithm of Oganov and co-workers^19^,^20^,^21^ to generate candidate crystal structures of the empirical formula BH_3_ for pressures of 30, 60, and 90 GPa. The only information used on input was that there are either 4 B and 12 H atoms or 6 B and 18 H atoms in a unit cell; no constraints were imposed on the type or number of bonds between the atoms. These runs produced a total of 5502 candidate structures. Out of these, we selected by inspection a short list of 134 candidates by keeping the structures that had either a low enthalpy or a high symmetry. To maintain diversity, we included comparable numbers of structures with two, three, or four B atoms per molecular unit (36, 18, and 21 structures, respectively), as well as 59 polymers of BH_3_ units. Then we optimized the lattice parameters and atomic coordinates of each of these 134 structures at the corresponding experimental pressure (36, 42, 57, 61, 74 and 88 GPa) using a modification of the Perdew–Burke–Ernzerhof (PBE)^22^ exchange-correlation functional known as “PBE for solids” (PBEsol)^23^. Finally, we calculated the Raman spectra of the candidate structures (see Methods for details).

**Figure 3:**
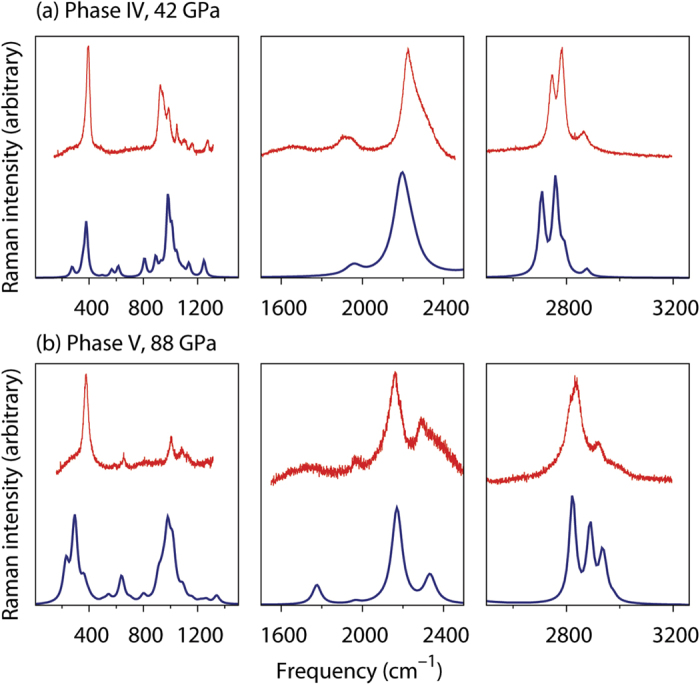
Comparison of experimental and simulated Raman spectra of phases IV and V. The experimental spectra (top, red) are the same as in Fig. 1. The simulated spectra (bottom, blue) are convoluted to Lorentzian lineshapes. Similar comparisons for other pressures are in the Supplementary Information.

By comparing the observed Raman spectra of phases IV and V to the simulated spectra of these 134 candidate structures, we found two best matches (Fig. 3) which correspond to the structures shown in Fig. 2b. The structure assigned to phase IV has a triclinic unit cell 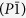 with two B_2_H_6_ units per cell (Z = 2), almost perpendicular to each other. The calculated cell parameters of phase IV at 42 GPa are *a* = 4.740 Å, *b* = 4.425 Å, *c* = 3.217 Å, *α* = 73.37°, *β* = 97.41°, and *γ* = 87.56°. The structure assigned to phase V also has a triclinic cell 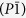 but with Z = 3; here the B_2_H_6_ molecules form a layered structure with B or H atoms roughly aligned in the [101] direction. The cell parameters of phase V at 88 GPa are *a* = 6.216 Å, *b* = 3.060 Å, *c* = 4.350 Å, *α* = 69.90°, *β* = 78.75°, and *γ* = 89.81°.

The simulated Raman spectra of phases IV and V exhibit all the essential features of the experimental profiles of Fig. 1, including a number of important details. In particular, the soft behavior of the lowest-frequency lattice mode is qualitatively reproduced by our calculations (Fig. 2a). The new modes near 600 and 2200 cm^−1^ at pressures of 57 GPa and higher (those labeled with asterisks in Fig. 1) are unambiguously present in the simulated spectra. Also reproduced are the complex features corresponding to the ring vibrations in the spectral region of 800–1300 cm^−1^ as well as the reversal of relative intensities of the terminal BH symmetric stretching modes near 2800 cm^−1^, which occurs between 42 and 57 GPa (Fig. 1). The experimental and simulated relative intensities of the dominant peaks near 2200 and 2400 cm^−1^ are consistent at both 42 and 88 GPa. Although only one prominent lattice mode is observed near 400 cm^−1^ at 88 GPa, the simulated spectra suggest that the broad base of this intense mode contains other lattice vibrations (Fig. 3b). All these facts strongly support the proposed structural assignments to phases IV and V. No other of the 134 crystal structures matched the experimental Raman spectra of phases IV and V nearly as well as the structures shown in Fig. 2b. Because the structure of phase V matches the experimental Raman spectra of compressed diborane not only at 57 GPa but also at every higher pressure (see Supplementary Section S3), we conclude that no other phase transitions occur between 57 and 88 GPa, and that diborane remains in molecular form up to at least 88 GPa.

**Figure 4:**
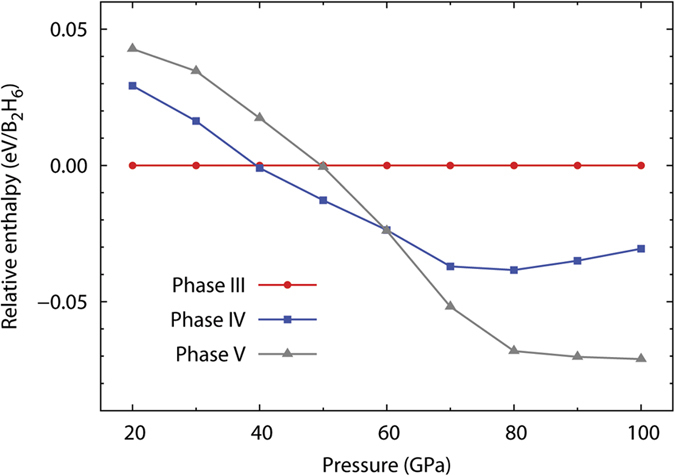
Calculated enthalpies of phases IV and V relative to phase III. All enthalpies are calculated using the PBEsol functional.

More evidence supporting our identification of phases III–V is provided by enthalpy calculations using the PBEsol functional. Although PBEsol^23^ was specifically developed to produce more accurate structural parameters than PBE at the expense of less accurate energies (at least at ambient pressure), we observed here and earlier in ref. 15 that the relative PBEsol enthalpies of various *high-pressure* polymorphs of diborane are nearly the same as those predicted with the PBE functional. According to PBEsol, phase III is the most stable among the three up to about 40 GPa, phase IV is the most stable from ~40 to ~60 GPa, and phase V is the most stable from ~60 up to at least 100 GPa (Fig. 4). The crossover points near 40 and 60 GPa are in good agreement with the experimentally deduced phase boundaries at 42 and 57 GPa. PBEsol phonon calculations for phase IV at 42 GPa and phase V at 57, 74, and 88 GPa revealed no imaginary frequencies at any of these pressures (see Supplementary Section S6). The absence of imaginary frequencies indicates that the proposed structures of phases IV and V are genuine minima. Interestingly, none of the phases III–V is the most stable thermodynamically among the 134 candidate structures at any pressure between 20 and 100 GPa. The candidate structure with the lowest enthalpy proved to be a *Pca*2_1_ phase of BH and H_2_, whose boron-containing units resemble the *Ibam* phase reported by Hu *et al*^16^. It is likely that dispersion interactions between diborane molecules also play a role in determining the relative stability of various polymorphs. However, because dispersion corrections usually rely on empirical short-range damping functions developed for systems where interatomic distances are not too short, we deemed it unsafe to apply those corrections to highly compressed structures.

**Figure 5:**
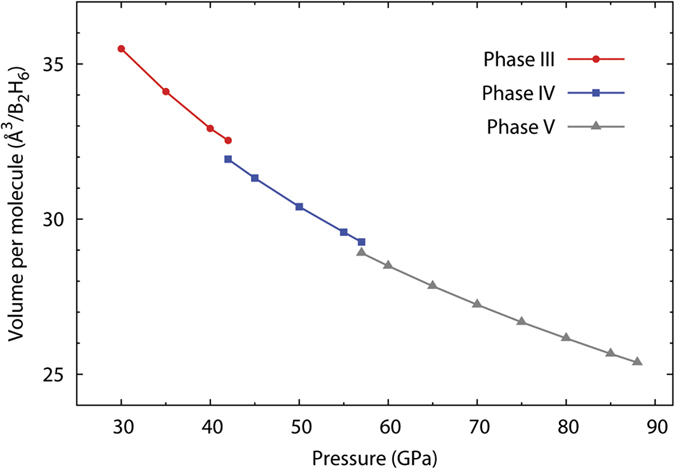
Calculated volume per B_2_H_6_ unit as a function of pressure. The volumes are calculated for the PBEsol structures optimized at a fixed pressure.

As an extra assurance of our structural assignments, we calculated and plotted the average volume per B_2_H_6_ unit for the proposed structures of phases III–V at various pressures. The resulting plots (Fig. 5) exhibit two volume contractions with ratios of 1.84% and 1.43% at 42 and 57 GPa, respectively, which are consistent with pressure-induced polymorphic transitions.

## Discussion

Our experiments and calculations strongly suggest that diborane remains in the molecular crystal form over an extended pressure range from 3.5 GPa to at least 88 GPa. At the same time, the soft behavior of the lattice mode in phase V hints at the possibility of upcoming structural changes at even higher pressures. Of particular interest in this regard are the two long-standing questions: 1) whether diborane eventually leaves the molecular B_2_H_6_ motif and forms polymeric boron hydride^14^; 2) whether sufficiently compressed diborane becomes metallic^13^.

**Figure 6:**
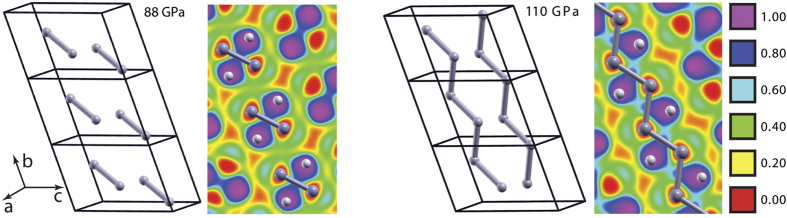
Units cells and ELF plots for phase V at 88 and 110 GPa. Units cells of phase V at 88 GPa (left) and 110 GPa (right) and the corresponding ELF plots are shown for the plane containing the right B-atom chain. The ELF is calculated using the HSE06 functional for the PBEsol-optimized structures. B–B bonds inside the unit cells are shown as sticks; H atoms inside the unit cells are removed for clarity.

To address the first of these questions, we explored the behavior of phase V at pressures above 88 GPa using the PBEsol method. We found that, between 88 GPa and 110 GPa, the crystal structure of phase V changes continuously, but still can be described as consisting of B_2_H_6_ molecules. Near 110 GPa, however, it finally leaves the molecular diborane motif and is best described as consisting of one-dimensional zigzag chains of boron atoms. This transformation is evidenced by the disappearance of B–B bond length alternation and by the absence of identifiable molecular units in plots of the electron localization function^24^ (Fig. 6). In particular, the B–B distances in zigzag chains along the b axis alternate between 1.605 Å and 2.084 Å at 88 GPa, whereas all B–B bond lengths in the chains are 1.716 Å at 110 GPa. Also shown in Fig. 6 are the corresponding plots of the electron localization function (ELF) for the plane containing a B atom chain. The areas with low ELF values represent the B atoms; the areas with high values represent the H atoms. Note that, at 88 GPa, there are two H atoms in the plane for every two B atoms, but at 138 GPa one of those H atoms moves out of the plane.

**Figure 7:**
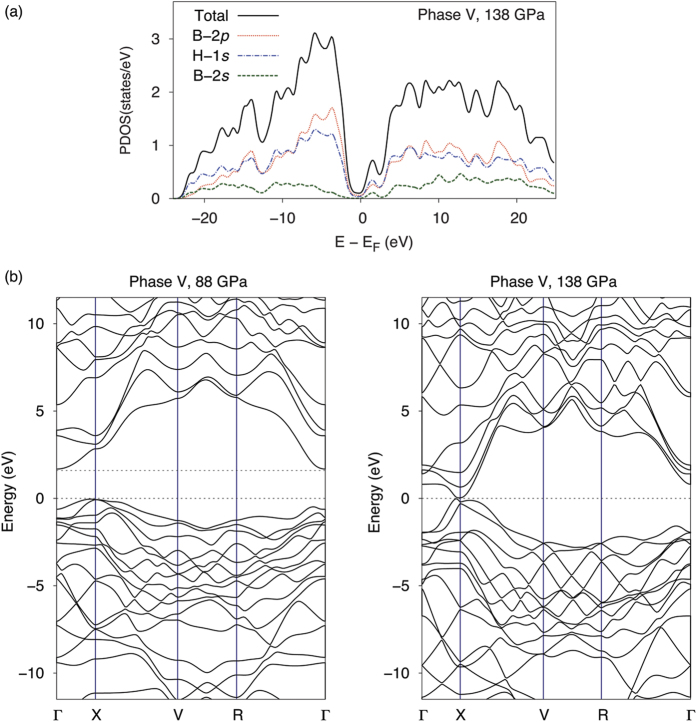
Densities of states and band structure of phase V. (**a**) Total and projected densities of states for phase V calculated at the predicted metallization pressure of 138 GPa. (**b**) Band structure of phase V calculated at 88 and 138 GPa. The unit cell parameters are optimized at each pressure using the PBEsol functional, the electronic energy bands are calculated using the HSE06 functional.

To investigate the possibility of pressure-induced metallization of compressed diborane, we calculated the bandgaps, the total density of states (DOS) and the projected DOS (PDOS) of phase V in the 36–200 GPa pressure range using the Heyd–Scuseria–Ernzerhof^25^,^26^,^27^ (HSE06) screened hybrid density functional, known for realistic bandgap predictions (Fig. 7). These calculations show that at 88 GPa, phase V is a semiconductor with a bandgap of 1.6 eV. The bandgap gradually decreases with increasing pressure and finally closes near 138 GPa, resulting in a nonzero total DOS at the Fermi level (Fig. 7). Analysis of the PDOS plots reveals that the main contribution to the metallization is from the boron’s 2*p* orbitals. Although extremely experimentally challenging, *in situ* conductivity measurements would be needed to characterize the electronic properties of diborane at high pressures in the future.

## Conclusion

In summary, we have reported and interpreted two previously unknown polymorphic transformations of compressed diborane occurring between 36 and 88 GPa. At the lower end of this pressure range, diborane exists in the form of phase III which transforms into phase IV near 42 GPa and then into phase V near 57 GPa. All these phases retain the diborane motif and are thermodynamically metastable. Future synchrotron and neutron-based *in situ* diffraction experiments should be able to verify these structure assignments and predictions. We also predict that phase V will remain in molecular form up to near-megabar pressures, above which it should transform into a structure with covalently bonded chains of boron atoms and eventually become metallic around 138 GPa. While such pressures have not yet been reached experimentally, they are feasible, which means that the first reports of pressure-induced polymerization and metallization of diborane could be a matter of time.

## Methods

Gaseous diborane of electronic grade (purity 99.99%) packed in a lecture bottle with a 10% concentration balanced with hydrogen was purchased from Aldrich and used without further purification. Sample loading was achieved cryogenically by precooling a diamond anvil cell in liquid nitrogen to below the melting point for B_2_H_6_, i.e., −165 °C. After gaseous B_2_H_6_ solidifies on the rhenium gasket, the cell was then sealed, and the solid B_2_H_6_ was pressurized before warming to room temperature. A few ruby chips were inserted for pressure measurements before the cryogenic loading. The pressure was determined from the well-established pressure shift of the R_1_ ruby fluorescence line with an accuracy of ±0.05 GPa under quasi-hydrostatic conditions. *In situ* Raman spectroscopy was used to monitor the structural changes as a function of pressure. The setup of the customized Raman microspectroscopy system and the measurement details have been described previously^12^.

All candidate crystal structures of diborane were generated using the evolutionary structure search algorithm as implemented in the USPEX code^19^,^20^,^21^. The enthalpies of the structures at 0 K were used as a fitness criterion: the 40% least stable structures were discarded and the remaining was allowed to produce the next generation through heredity (60%), soft-mutation (20%) and lattice mutation (20%). Two distinct lowest-enthalpy structures of each generation were allowed to survive into the subsequent one, and the runs were terminated after 50 generations. The enthalpy calculations were performed with the Quantum ESPRESSO program (version 5.0.1)^28^. Because Raman spectral intensities are implemented in Quantum ESPRESSO only for the local density approximation (LDA), we re-optimized all structures at the LDA level and calculated Raman frequencies and intensities using the LDA. The resulting vibrational frequencies were used without applying any scale factors. The pseudopotentials were taken from the Quantum ESPRESSO pseudopotential library. In the PBEsol calculations, PBE ultrasoft Vanderbilt pseudopotentials with a cutoff energy of 90 Ry were used. In the LDA calculations, the norm-conserving LDA pseudopotentials with a cutoff energy of 130 Ry were employed. The Brillouin zone was sampled using the homogeneous Monkhorst–Pack **k**-point meshes with reciprocal space resolution of 0.08π Å^−1^. The convergence criterion for Kohn–Sham self-consistency cycles was set to 10^−10^ Ry. All structure optimizations were performed using the Broyden–Fletcher–Goldfarb–Shanno quasi-Newton method. The optimization was stopped when the components of all Hellmann–Feynman forces dropped below 10^−4^ Ry/Å and the stress on the cell was within 0.05 GPa of the target. The phonon frequency was obtained by diagonalization of the dynamical matrix calculated by the density-functional perturbation theory. The non-resonant Raman intensities were computed from the second-order derivative of the electronic density matrix with respect to a uniform electric field.

## Additional Information

**How to cite this article**: Torabi, A. *et al.* Polymorphic transitions of diborane at sub- and near-megabar pressures. *Sci. Rep.* **5**, 13929; doi: 10.1038/srep13929 (2015).

## Supplementary Material

Supplementary Information
