# Supplementary material for: Polymorphic transitions of diborane at sub- and near-megabar pressures
Source: Sci Rep. 2015 Sep 10;5:13929. doi: 10.1038/srep13929 (PMC4564767; doi:10.1038/srep13929)
Supplement: Supplementary Information [file srep13929-s1.pdf]

SUPPLEMENTARY INFORMATION

Polymorphic transitions of diborane at sub- and near-megabar pressures

Amin Torabi, Chitra Murli, Yang Song, and Viktor N. Staroverov

Contents

Section S1 Calculated structural parameters of phase IV

Section S2 Calculated structural parameters of phase V

Section S3 Experimental and simulated Raman spectra of phases III and V

Section S4 Volume per B_2H_6 unit for phases III–V as a function of pressure

Section S5 Calculated bandgaps of phases III–V as functions of pressure

Section S6 Calculated phonon dispersion plots for phases IV and V

Section S7 The 134 shortlisted candidate structures of boron hydride

Section S1. Cell parameters, atomic positions in fractional crystal coordinates, and Wyckoff positions for the phase IV structure optimized at 42 GPa using the PBEsol functional.

Phase IV ($P\bar{1}$, $Z = 2$), 42 GPa

a, b, c (Å)	4.740	4.425	3.217
α, β, γ (deg)	73.37	97.41	87.56
B 2i	0.1197	0.4017	−0.0853
B 2i	−0.3613	0.0929	−0.4312
H 2i	0.3363	−0.4928	−0.1698
H 2i	−0.1483	0.0399	0.4453
H 2i	0.0697	0.1588	−0.1466
H 2i	−0.3850	0.2769	−0.2375
H 2i	−0.4614	−0.1610	−0.2108
H 2i	−0.0921	−0.3944	−0.3085

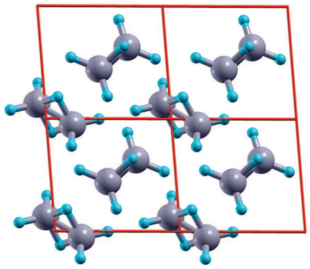

Section S2. Cell parameters, atomic positions in fractional crystal coordinates, and Wyckoff positions for the phase V structures optimized at 57, 61, 74, and 88 GPa using the PBEsol functional.

Phase V ($P\bar{1}$, $Z = 3$), 57 GPa

a, b, c (Å)	6.497	3.185	4.550
α, β, γ (deg)	69.75	79.03	89.90
B 2i	0.2762	−0.0297	0.4463
B 2i	−0.3927	0.2869	−0.2133
B 2i	−0.0617	−0.2348	0.1228
H 2i	−0.4483	−0.4426	0.2587
H 2i	−0.1120	−0.1035	−0.4090
H 2i	0.3553	−0.0030	−0.3496
H 2i	−0.3118	0.3093	−0.0106
H 2i	−0.2598	0.4424	−0.4742
H 2i	−0.4080	−0.1305	−0.1787
H 2i	−0.2236	−0.3382	0.0836
H 2i	0.0690	−0.1569	−0.1391
H 2i	0.0187	−0.4611	0.3252

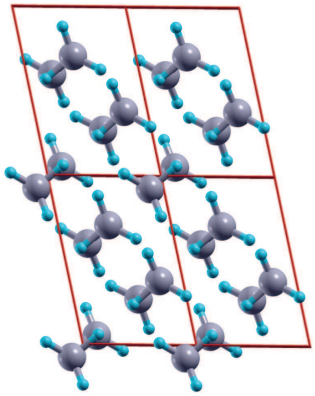

Phase V ($P\bar{1}$, $Z = 3$), 61 GPa

a, b, c (Å)	6.455	3.168	4.519
α, β, γ (deg)	69.75	78.97	89.88
B 2i	0.2765	−0.0271	0.4460
B 2i	−0.3927	0.2879	−0.2135
B 2i	−0.0619	−0.2352	0.1232
H 2i	−0.4477	−0.4454	0.2592
H 2i	−0.1112	−0.1046	−0.4084
H 2i	0.3557	−0.0001	−0.3487
H 2i	−0.3110	0.3091	−0.0098
H 2i	−0.2587	0.4422	−0.4761
H 2i	−0.4091	−0.1322	−0.1765
H 2i	−0.2245	−0.3390	0.0836
H 2i	0.0696	−0.1578	−0.1404
H 2i	0.0189	−0.4623	0.3264

Phase V ($P\bar{1}$, $Z = 3$), 74 GPa

a, b, c (Å)	6.330	3.112	4.431
α, β, γ (deg)	69.79	78.91	89.85
B 2i	0.2777	−0.0152	0.4439
B 2i	−0.3919	0.2928	−0.2152
B 2i	−0.0628	−0.2367	0.1246
H 2i	−0.4461	−0.4533	0.2609
H 2i	−0.1087	−0.1099	−0.4059
H 2i	0.3570	0.0074	−0.3451
H 2i	−0.3083	0.3084	−0.0077
H 2i	−0.2556	0.4384	−0.4810
H 2i	−0.4125	−0.1356	−0.1701
H 2i	−0.2275	−0.3414	0.0836
H 2i	0.0701	−0.1610	−0.1424
H 2i	0.0200	−0.4646	0.3301

Phase V ($P\bar{1}$, $Z = 3$), 88 GPa

a, b, c (Å)	6.216	3.060	4.350
α, β, γ (deg)	69.90	78.75	89.81
B 2i	0.2799	0.0006	0.4404
B 2i	−0.3907	0.2988	−0.2172
B 2i	−0.0644	−0.2369	0.1269
H 2i	−0.4446	−0.4599	0.2641
H 2i	−0.1063	−0.1142	−0.4040
H 2i	0.3580	0.0118	−0.3404
H 2i	−0.3050	0.3064	−0.0059
H 2i	−0.2520	0.4311	−0.4862
H 2i	−0.4167	−0.1381	−0.1613
H 2i	−0.2305	−0.3434	0.0833
H 2i	0.0701	−0.1663	−0.1438
H 2i	0.0209	−0.4653	0.3334

Section S3. Comparison of experimental (red) and simulated (blue) Raman spectra of phases III and V at selected pressures. This figure complements Fig. 3 of the main text.

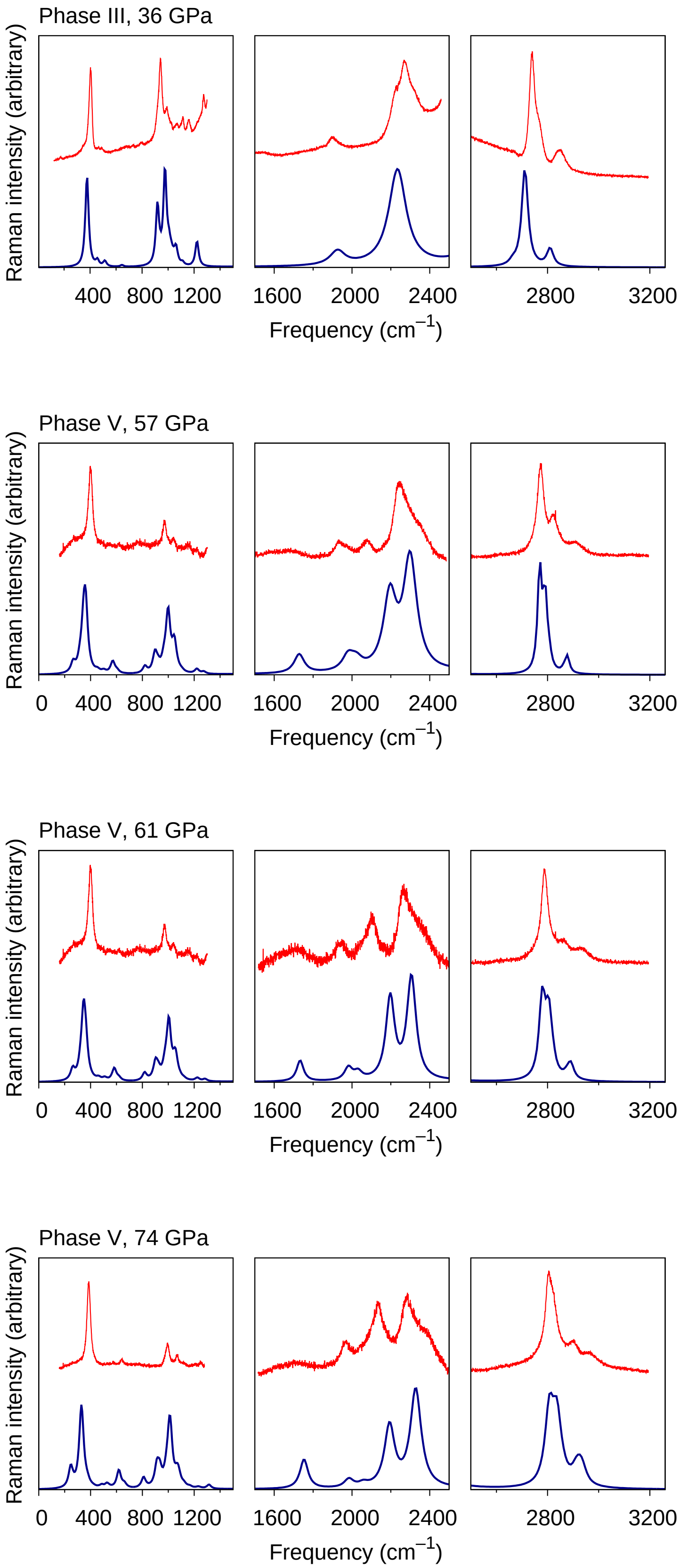

Section S4. Calculated volume per B_2H_6 unit for the PBEsol structures of phases III–V as a function of pressure.

Phase III

Pressure (GPa)	Volume ($Å^3/B_2H_6$)
30	35.49
35	34.11
40	32.92
42	32.54
45	31.88
50	30.95
55	30.12
57	29.82
60	29.37
65	28.68
70	28.04
75	27.44
80	26.88
85	26.37
88	26.02

Phase IV

Pressure (GPa)	Volume ($Å^3/B_2H_6$)
30	34.90
35	33.55
40	32.36
42	31.94
45	31.32
50	30.40
55	29.57
57	29.26
60	28.82
65	28.12
70	27.44
75	26.08
80	25.58
85	25.12
88	24.87

Phase V

Pressure (GPa)	Volume ($Å^3/B_2H_6$)
30	34.15
35	32.84
40	31.74
42	31.34
45	30.76
50	29.90
55	29.12
57	28.84
60	28.42
65	27.77
70	27.18
75	26.63
80	26.11
85	25.62
88	25.34

Section S5. HSE06 bandgaps of the PBEsol structures of phases III–V calculated at various pressures.

Phase III

Pressure (GPa)	Bandgap (eV)
36	4.20
42	3.94
57	3.43
61	3.31
74	2.93
88	2.54
100	2.23
110	1.99
120	1.78
130	1.51
140	1.23
150	0.96
160	0.73
180	0.22
200	−0.26

Phase IV

Pressure (GPa)	Bandgap (eV)
36	3.97
42	3.75
57	3.31
61	3.19
74	2.81
88	2.66
100	2.44
110	2.27
120	2.11
130	1.95
140	1.79
150	1.64
160	1.49
180	1.21
200	0.95

Phase V

Pressure (GPa)	Bandgap (eV)
36	3.15
42	2.92
57	2.41
61	2.29
74	1.93
88	1.58
100	1.31
110	0.75
120	0.48
130	0.17
137	0.01
138	−0.05
140	−0.15
150	−0.42
200	−0.73

Section S6. Phonon dispersion plots for phases IV and V calculated using the PBEsol functional.

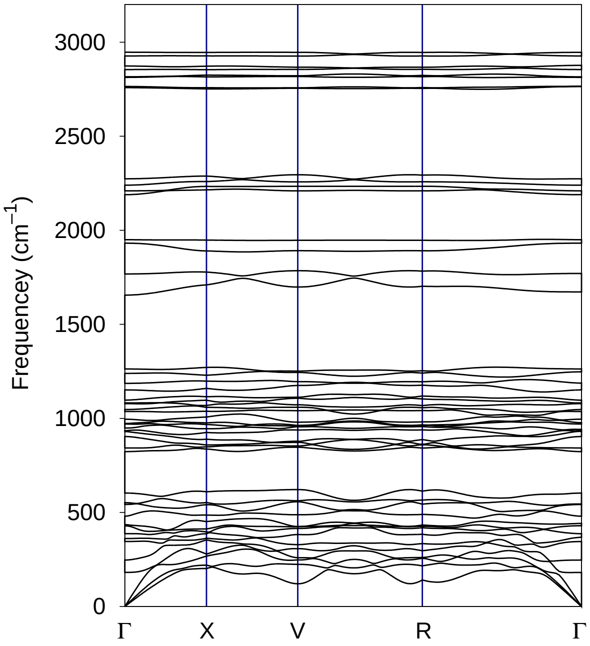

Phase IV, 42 GPa

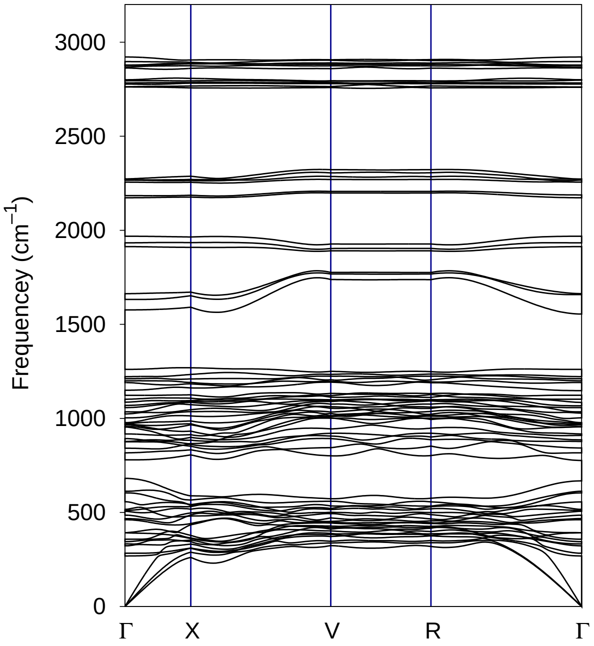

Phase V, 57 GPa

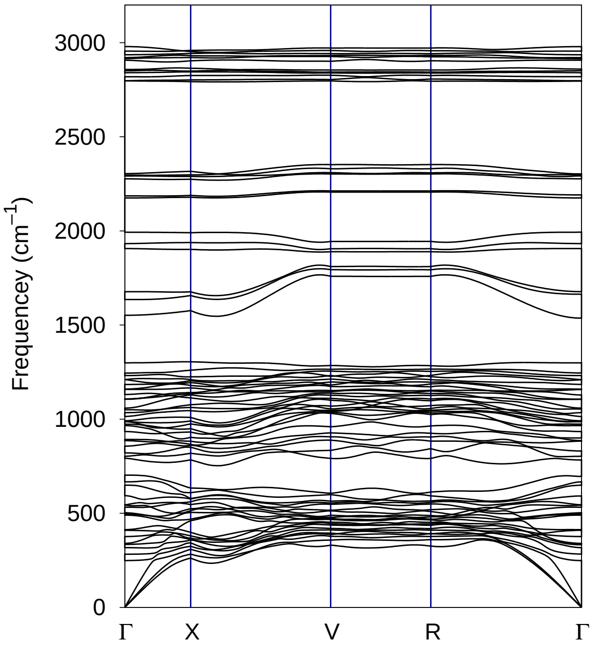

Phase V, 74 GPa

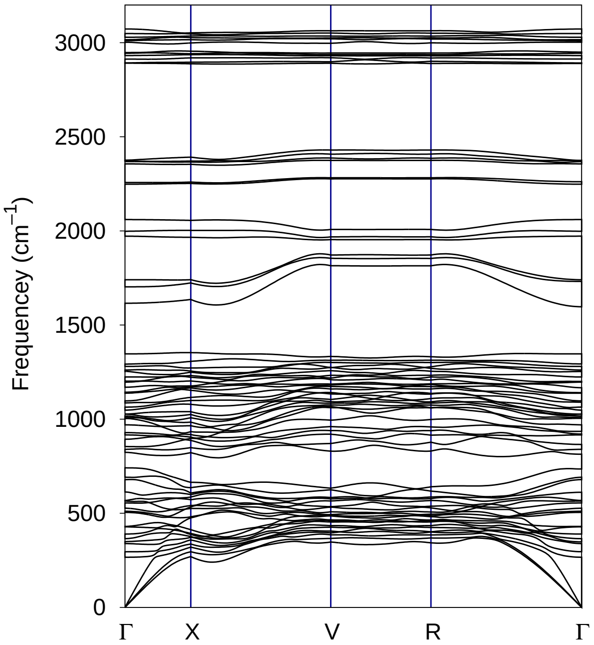

Phase V, 88 GPa

==
Section 7. The 134 shortlisted structures of boron hydride.

These were selected from among 5502 candidate structures generated by the USPEX program. All structures are shown in the POSCAR as they appeared in the output of the USPEX program. For an explanation of the POSCAR format, see http://cms.mpi.univie.ac.at/vasp/guide/node59.html
==

```
EA1 3.77 3.59 6.11 89.7 105 63.5
1.0
3.7662827747 0 0
1.6051512751 3.2133116405 0
-1.5956278277 0.83697372851 5.8358131908
4 12
Direct
0.102576368 0.876208766 0.979529964
0.398458685 0.118821018 0.019731209
0.599978084 0.477696389 0.574074307
0.901110042 0.517317866 0.425198024
0.973117937 0.803054067 0.443041901
0.528010783 0.191936223 0.556159496
0.035494793 0.776348422 0.141505392
0.465492216 0.218625642 0.857720004
0.005732523 0.304018287 0.6199681
0.49539338 0.691145683 0.379342467
0.996713945 0.270312688 0.967536942
0.504316203 0.724702507 0.031798779
0.992649338 0.28753877 0.291658099
0.508407563 0.707410632 0.707640944
0.506510186 0.197341326 0.202387647
0.994578511 0.79772896 0.796855767

EA2 4.86 3.39 4.61 106 89.1 91.5
1.0
4.8557775148 0 0
-0.086688763718 3.3861965752 0
0.076355032842 -1.2448484679 4.4394131415
4 12
Direct
0.041025589 0.480831009 0.673301744
0.964191523 0.512291515 0.33013477
0.545386844 0.897173027 0.823888067
0.45970515 0.096282473 0.179360954
0.00490047 0.238293023 0.118192228
0.467225333 0.563954384 0.70597634
0.678799264 0.084172088 0.693921423
0.875699301 0.818548629 0.308394995
0.000621864 0.754434672 0.885580204
0.537356998 0.429268891 0.298683432
0.32597661 0.907485961 0.30770497
0.129247724 0.174186596 0.694590162
0.188148594 0.601074869 0.478969778
0.816930312 0.392597041 0.524656187
0.665585645 0.872127831 0.062871213
0.340523894 0.123581509 0.940375275

EA3 5.79 3.82 3.36 96 103 85.2
1.0
5.7867737633 0 0
0.31732011787 3.8022822155 0
-0.73467417506 -0.2932889054 3.2691412844
4 12
Direct
0.382276495 0.127159232 0.839589066
0.613686133 0.871699495 0.727359991
0.873515012 0.401752379 0.266439524
```

```
0.122467896 0.597187003 0.300300418
0.503116313 0.1523635 0.567387707
0.578555539 0.639872926 0.468993898
0.713687875 0.475191389 0.005232647
0.049804998 0.306746119 0.095615037
0.807412797 0.947438365 0.872320208
0.188602893 0.051596738 0.693903141
0.138723879 0.799739835 0.072805988
0.857427664 0.199232886 0.494051045
0.492726138 0.846474393 0.999566056
0.417345534 0.35926555 0.097642124
0.28227229 0.52346079 0.561418843
0.945930358 0.692256544 0.470807777

EA4 4.66 4.86 3.36 89.8 71.9 90
1.0
4.6637014014 0 0
-0.0015678325521 4.8577223013 0
1.0397813205 0.011095114856 3.1902933016
4 12
Direct
0.080694276 0.051666995 0.189435919
0.580353541 0.449839269 0.688700062
0.919341899 0.549017097 0.689324186
0.418257089 0.947619152 0.18978021
0.434817706 0.80102688 0.457507631
0.737363609 0.658901895 0.516449289
0.065319465 0.19836058 0.921107051
0.561970046 0.304654403 0.421808915
0.864852374 0.9870329 0.458880685
0.366452543 0.515432011 0.95951473
0.762470359 0.339832687 0.861390746
0.233937058 0.841094074 0.01830626
0.937646302 0.69445009 0.955955502
0.633671945 0.012314554 0.919637693
0.264015702 0.156994929 0.363702519
0.13340389 0.483508976 0.4186851

EA5 3.47 3.43 5.92 89.2 91.5 89.3
1.0
3.4725729547 0 0
0.042876596852 3.432458037 0
-0.15049511408 0.079674559933 5.9202366556
4 12
Direct
0.885468466 0.918780449 0.379719837
0.063427674 0.571424899 0.552651392
0.38524746 0.918779829 0.879726488
0.563380251 0.571498985 0.0526528
0.371855369 0.446781028 0.19563769
0.877444957 0.454937876 0.029188425
0.077120526 0.043398307 0.236766437
0.877131788 0.545430659 0.362267022
0.376954903 0.545393939 0.862333947
0.071884902 0.944776717 0.570058788
0.571439245 0.035489655 0.40316437
0.87179098 0.446808332 0.695617642
0.57686747 0.043456066 0.736765208
0.071168901 0.035371935 0.903144481
0.377483954 0.454758452 0.529248786
0.571535464 0.944856929 0.070108544

EA6 3.47 3.43 5.92 89.2 91.5 89.3
1.0
3.4725729547 0 0
0.042876596852 3.432458037 0
-0.15049511408 0.079674559933 5.9202366556
```

4 12
Direct
0.885468466 0.918780449 0.379719837
0.063427674 0.571424899 0.552651392
0.38524746 0.918779829 0.879726488
0.563380251 0.571498985 0.0526528
0.371855369 0.446781028 0.19563769
0.877444957 0.454937876 0.029188425
0.077120526 0.043398307 0.236766437
0.877131788 0.545430659 0.362267022
0.376954903 0.545393939 0.862333947
0.071884902 0.944776717 0.570058788
0.571439245 0.035489655 0.40316437
0.87179098 0.446808332 0.695617642
0.57686747 0.043456066 0.736765208
0.071168901 0.035371935 0.903144481
0.377483954 0.454758452 0.529248786
0.571535464 0.944856929 0.070108544

EA7 3.54 3.78 5.41 90 90 83.1
1.0
3.5445405062 0 0
0.4558246571 3.7519548954 0
0.00083463258151 -0.0018542126716 5.412008445
4 12
Direct
0.364498327 0.618385358 0.577843255
0.606774028 0.224025114 0.63692402
0.109194178 0.227230538 0.077983902
0.867320135 0.621972673 0.136795119
0.376186634 0.122852164 0.194149678
0.315955465 0.306441494 0.497194865
0.097381615 0.722574038 0.694014605
0.512507491 0.056213831 0.802395229
0.601676156 0.722515718 0.018381913
0.158610739 0.539081594 0.997530156
0.651697998 0.535089191 0.720731428
0.010919531 0.063318562 0.911475335
0.462778059 0.782255844 0.411384151
0.872755599 0.123255164 0.518791237
0.822021329 0.31122697 0.220918042
0.960941852 0.790257126 0.302252157

EA8 3.47 3.43 5.92 89.2 91.5 89.4
1.0
3.4703446081 0 0
0.038638957086 3.4342593294 0
-0.15578274156 0.081991409985 5.9215601136
4 12
Direct
0.199913921 0.612999905 0.512181042
0.376079554 0.264864885 0.685085167
0.699392007 0.611630428 0.012126597
0.876948191 0.264422418 0.185224582
0.685163059 0.140621598 0.328358614
0.392279205 0.736132919 0.369154736
0.188202596 0.239941338 0.495092653
0.689620911 0.238377207 0.995111338
0.886496252 0.731491653 0.535724925
0.385625014 0.728936689 0.035821515
0.689530313 0.146807196 0.661203016
0.886563428 0.637599127 0.202319342
0.891014819 0.735590827 0.868955522
0.387107457 0.637930451 0.702390507
0.184317108 0.141237678 0.828263412
0.190753116 0.147117886 0.161671272

```
EA9 3.37 4.57 4.92 88.1 84.1 106
1.0
3.3705316826 0 0
-1.2563394326 4.3953990194 0
0.50789664897 0.31233101821 4.8885666951
4 12
Direct
0.078961769 0.402591937 0.121574411
0.438300178 0.0924968 0.635384498
0.919424132 0.593602876 0.883631514
0.561499728 0.903773813 0.370083245
0.133316936 0.50295263 0.334241179
0.532913273 0.032449319 0.846893924
0.865899179 0.492838636 0.671022576
0.465810681 0.964411153 0.159072624
0.147097349 0.170732303 0.075171198
0.265456488 0.277594288 0.607070968
0.850955953 0.825534579 0.929265057
0.734616693 0.718692118 0.39744586
0.226032688 0.848904573 0.535038167
0.289496698 0.60448305 0.931263457
0.774589743 0.146340566 0.471305054
0.708816626 0.392572527 0.074623981

EA10 3.59 3.58 5.35 90 90 90.2
1.0
3.587274988 0 0
-0.012112095264 3.5765143815 0
-0.00023417727688 -0.00019184896665 5.3546820306
4 12
Direct
0.481072493 0.5179855 0.275136576
0.725443739 0.235732299 0.526589645
0.766041025 0.766500491 0.026499147
0.010499316 0.484404756 0.775064929
0.487819475 0.815894429 0.916018248
0.42803593 0.239368613 0.164614436
0.063270655 0.762965387 0.664425387
0.003519866 0.185901597 0.416018904
0.513216493 0.522349903 0.508862031
0.52711271 0.980484643 0.559677916
0.963934868 0.022058381 0.059671361
0.978829309 0.480298798 0.00877911
0.230528138 0.72126042 0.306519921
0.718137043 0.278853297 0.759759557
0.773729007 0.723000197 0.259581851
0.261181385 0.281341215 0.806559468

EA11 3.72 5.37 3.53 90 90 85.8
1.0
3.7176637878 0 0
0.3891566898 5.3523169404 0
0.00015775410359 -0.0001104189841 3.5299823468
4 12
Direct
0.303985049 0.656614793 0.522331907
0.691608961 0.533293002 0.34162565
0.691478197 0.03319916 0.02222859
0.303841024 0.156697821 0.841723262
0.763590779 0.56454859 0.024235091
0.114810856 0.286370368 0.035726078
0.880270252 0.402968766 0.535016211
0.620435535 0.242853286 0.852240687
0.88066802 0.903413475 0.82854642
0.76279523 0.063876239 0.33992681
0.232263362 0.625216379 0.839757723
0.374852449 0.447377498 0.35117191
```

0.620647961 0.74265071 0.512410227
0.374977542 0.946925816 0.011335284
0.115164563 0.787049671 0.329257356
0.232241362 0.126172204 0.524064526

EA12 3.58 3.58 5.35 90 90 90
1.0
3.5800669007 0 0
0.001617101536 3.5815458165 0
1.7893905077e-05 2.9403778765e-05 5.3485265298
4 12
Direct
0.233995809 0.738066395 0.277478024
0.516767821 0.984309992 0.527717286
0.98698338 0.021316668 0.027708857
0.269751589 0.267543969 0.777468187
0.991372357 0.318633967 0.666287453
0.93653649 0.74286328 0.916606508
0.567175357 0.262771441 0.416610203
0.512379423 0.686949334 0.16630173
0.22724872 0.773861538 0.511466985
0.470789146 0.520689578 0.808650565
0.032938079 0.484927536 0.308644341
0.276520024 0.231728161 0.011451402
0.770841917 0.784388127 0.55926552
0.478475097 0.976751284 0.761585007
0.025266428 0.028852958 0.261575711
0.732932672 0.22127523 0.059242019

EA13 3.47 3.43 5.92 89.2 91.5 89.3
1.0
3.4711957204 0 0
0.039646468479 3.4334285245 0
-0.15363138782 0.081179391957 5.9212231077
4 12
Direct
0.343226235 0.333394942 0.493354572
0.520442924 0.985863349 0.666343936
0.020876065 0.986055421 0.166317607
0.843272173 0.333454292 0.993352397
0.535048285 0.457322393 0.35025746
0.333322266 0.960140167 0.476186077
0.029483989 0.450817265 0.516963689
0.52950812 0.450691896 0.017032396
0.03052592 0.359340658 0.18345886
0.833892361 0.960230647 0.97607938
0.530290169 0.359081471 0.683562359
0.328650484 0.861942171 0.809434823
0.334689573 0.868855032 0.142699722
0.829160202 0.861953558 0.309420789
0.834118727 0.868313866 0.6427095
0.034876364 0.457601046 0.850227402

EA14 6.36 3.49 3.44 91.1 104 74.3
1.0
5.4070868945 3.3532619178 0
-0.96867694553 3.3532619178 0
-0.81286583522 -0.30324334396 3.3269183855
4 12
Direct
0.620566087 0.911939802 0.935840342
0.441396505 0.151958251 0.19702556
0.111387293 0.671180173 0.522716638
0.951755739 0.392325985 0.611126002
0.068558128 0.82319425 0.202371604
0.759909518 0.059923213 0.901567085
0.996611357 0.238458313 0.930954553

0.935531947 0.762619325 0.664361115
0.629098117 0.879419877 0.310885368
0.302106792 0.004185019 0.230911353
0.265778329 0.702375894 0.776860431
0.797436935 0.359248501 0.357962404
0.459656138 0.451262941 0.343676566
0.601500591 0.613041613 0.788583463
0.128220743 0.300054716 0.472331982
0.431559086 0.182249706 0.821988149

EA15 3.41 3.42 5.9 90 90 90.6
1.0
3.4103916902 0 0
-0.035337129768 3.4241573696 0
-0.0034403722744 4.922359764e-05 5.8953244787
4 12
Direct
0.328535647 0.757371549 0.108368844
0.085720315 0.976857028 0.357549415
0.505604623 0.057035935 0.857657676
0.329012101 0.757287974 0.606767509
0.946456712 0.665180079 0.357525041
0.658680528 0.858252603 0.013417243
0.065129551 0.613073704 0.012348229
0.837158384 0.213330663 0.357697528
0.530631711 0.531641888 0.201950125
0.744117426 0.309124136 0.857858638
0.659070069 0.858184997 0.702149287
0.195014608 0.192112271 0.857320123
0.298599496 0.105662277 0.513342885
0.531150264 0.531506127 0.513396672
0.065405858 0.613111902 0.702495724
0.29746239 0.105948873 0.201447914

EA16 6.39 3.47 3.44 90.5 104 74.4
1.0
6.3912141766 0 0
0.93575740066 3.3411633799 0
-0.85149865263 0.20673205008 3.3305882739
4 12
Direct
0.039530953 0.007486405 0.454335486
0.710224327 0.515918155 0.778981573
0.546759333 0.24635197 0.875800266
0.217486227 0.754808253 0.200419514
0.672101921 0.658389236 0.455152245
0.584850756 0.103900207 0.199629981
0.530043383 0.621438307 0.904609
0.893304102 0.874893631 0.477289797
0.859128725 0.560153737 0.036505175
0.397861388 0.20217696 0.61822234
0.065400887 0.300291805 0.606373526
0.191634338 0.461994959 0.048375989
0.726963636 0.140819313 0.750241505
0.035730477 0.041490731 0.082830166
0.363689312 0.887478833 0.177499663
0.22129894 0.720793167 0.571911162

EA17 4.95 3.19 4.95 71.3 104 109
1.0
3.9202400725 -3.0200636935 0
1.0347249129 3.0200636935 0
-0.20890460776 1.7536979204 4.627205002
4 12
Direct
0.216944005 0.592703415 0.788545643
0.545236778 0.516291699 0.448943975

0.695223073 0.164883542 0.299363905
0.036371706 0.09005403 0.971453271
0.755665748 0.336292886 0.512496627
0.05040635 0.492283804 0.977451513
0.645820944 0.380265752 0.060708541
0.099015128 0.739832706 0.558557806
0.935623376 0.138513508 0.356864981
0.442638561 0.80810965 0.877454584
0.224908491 0.190660963 0.80458376
0.485364383 0.839853311 0.235410385
0.126913339 0.876750838 0.196998099
0.605145686 0.54786855 0.688227098
0.806350399 0.940035484 0.854557139
0.305446249 0.303532575 0.401675943

EA18 3.65 5.34 3.67 93.5 90 90
1.0
3.64977204 0 0
0.00021326894356 5.3361829663 0
-0.00027115250407 -0.22153934165 3.662152352
4 12
Direct
0.281853712 0.355644797 0.777891881
0.01768758 0.85560576 0.277894303
0.517402514 0.435185067 0.158469246
0.782176873 0.935201027 0.658432722
0.555956772 0.506448085 0.831221487
0.243009295 0.284659323 0.105293337
0.024877798 0.467786999 0.692309898
0.743465954 0.006304308 0.331123294
0.056498178 0.784619039 0.605283205
0.525291627 0.822953202 0.743865773
0.379159795 0.181312159 0.595274265
0.274588137 0.967812844 0.192370994
0.420544389 0.609517546 0.341337692
0.774303018 0.322863974 0.243757113
0.879135246 0.109556935 0.841176594
0.920512396 0.681246199 0.095269557

EA19 3.47 5.93 3.44 90.9 89.6 88.4
1.0
3.4669023586 0 0
0.16067653673 5.9259118454 0
0.024984341856 -0.054419929167 3.4369525171
4 12
Direct
0.198651968 0.417857674 0.328658367
0.698609883 0.917848701 0.328453927
0.371425249 0.244530024 0.979767825
0.871366402 0.744509245 0.979548363
0.391028764 0.562016941 0.448215491
0.88672496 0.393506192 0.449981852
0.680981588 0.933634585 0.955713794
0.888930634 0.728703565 0.352298095
0.386628999 0.89352796 0.449648601
0.389221118 0.228792584 0.352512876
0.180964627 0.43363683 0.955918775
0.890994645 0.061968085 0.44818614
0.183330285 0.76882956 0.858290186
0.179111893 0.100354247 0.860146205
0.678940125 0.600388503 0.859890505
0.683368775 0.268876766 0.858493834

EA20 3.42 3.41 6.06 90.2 89.6 91
1.0
3.420943306 0 0
-0.059750683032 3.4063662251 0

0.042299045231 -0.020035140061 6.0602561756
4 12
Direct
0.828676669 0.552487081 0.22355229
0.175186534 0.401532913 0.721813093
0.829050804 0.052192165 0.723455966
0.174660616 0.902003599 0.221830598
0.703954262 0.435699926 0.055985156
0.290733358 0.526011365 0.553482092
0.704767863 0.934437351 0.55598761
0.288910347 0.026666499 0.053295893
0.713699808 0.92805795 0.891908722
0.299878774 0.019400827 0.389155682
0.713849142 0.427323267 0.391873193
0.299349252 0.518980772 0.889368144
0.799851922 0.929474307 0.227951831
0.203937767 0.024509803 0.71817654
0.800347601 0.429236221 0.72688252
0.203531027 0.525021116 0.218356576

EA21 3.39 3.48 6.02 91.2 90 90
1.0
3.3868654136 0 0
0.0006748433287 3.4779655109 0
0.00096355347177 -0.12092217206 6.0167818443
4 12
Direct
0.560174305 0.675145523 0.185934255
0.225142041 0.8501454 0.362418467
0.725097607 0.173854076 0.68615347
0.059795693 0.349493515 0.862532018
0.111168486 0.657782809 0.506288918
0.674584183 0.867450718 0.042171539
0.174196157 0.157111155 0.006250342
0.10415193 0.16220397 0.339146809
0.680878104 0.362990231 0.209075978
0.181953859 0.661399031 0.17820533
0.103374441 0.160923307 0.678303239
0.18071313 0.661498052 0.839282047
0.610276073 0.365200261 0.542014304
0.605106623 0.861648222 0.709872303
0.603409645 0.864177628 0.370004824
0.681504359 0.364012237 0.86992906

EA22 4.47 3.46 4.97 99.2 72.6 103
1.0
4.4707103565 0 0
-0.75869020061 3.3760896163 0
1.4899361937 -0.47847640963 4.7193806729
4 12
Direct
0.827152089 0.261750379 0.302123878
0.491945471 0.2779076 0.206634391
0.843170177 0.421820019 0.98118792
0.54682691 0.097020427 0.851461701
0.859397112 0.534432082 0.467578869
0.329685521 0.509616171 0.32248931
0.910008957 0.976581456 0.330749972
0.875699077 0.194178466 0.757068343
0.523740415 0.069019394 0.375290561
0.420032321 0.294579987 0.766392483
0.912367602 0.752988626 0.940457652
0.353609427 0.957879154 0.096636453
0.55029106 0.772672282 0.734356113
0.169894374 0.842898123 0.632525587
0.057933677 0.310228728 0.04270918
0.123470149 0.935312116 0.529554549

EA23 4.45 4.4 4.66 90.5 117 115
1.0
4.4548040862 0 0
-1.8571243733 3.9863757772 0
-2.0908356056 -1.0147389183 4.0425590919
4 12
Direct
0.295037415 0.341257344 0.363222364
0.82660963 0.207243962 0.263890146
0.921764326 0.226689363 0.956753184
0.818899826 0.536387095 0.060687375
0.4982555 0.642480463 0.453360276
0.110001566 0.294878862 0.54177951
0.430456666 0.165056353 0.468257109
0.062552755 0.865923105 0.698698842
0.791377546 0.458817659 0.33327846
0.081318922 0.80916094 0.205921198
0.256887847 0.306577592 0.049850196
0.715464721 0.972248477 0.747014614
0.945079607 0.489907492 0.852780967
0.527076388 0.529502785 0.890056067
0.239124413 0.868846939 0.718639411
0.580816867 0.93615229 0.221965421

EA24 3.66 4.7 4.7 80.3 67.3 67.2
1.0
3.6622754333 0 0
1.8184291228 4.3356424486 0
1.8115309592 0.098535243611 4.337957618
4 12
Direct
0.835861046 0.370819799 0.617424552
0.163858108 0.599777527 0.347392848
0.830408156 0.876328539 0.123195249
0.116755739 0.146711193 0.894107806
0.854156976 0.413353438 0.868188658
0.139040872 0.625143585 0.081211815
0.902271707 0.103247342 0.171560697
0.371451039 0.962768787 0.711831289
0.535068899 0.504295595 0.238422196
0.181057434 0.322090685 0.391236954
0.287002412 0.25516423 0.988326726
0.78732645 0.15858059 0.559692634
0.786025812 0.827365229 0.904576644
0.514833176 0.934553078 0.335765386
0.056301053 0.782327597 0.530789945
0.526924463 0.590252811 0.666318412

EA25 4.84 3.42 4.84 70.2 77.6 70.1
1.0
4.8418143932 0 0
1.1673183672 3.216524596 0
1.0422315714 1.3707590674 4.5279098354
4 12
Direct
0.866159937 0.868047398 0.86222033
0.366223865 0.36822733 0.362294611
0.694609625 0.685716585 0.690594409
0.194764936 0.185624433 0.190804488
0.093114541 0.931587375 0.753390928
0.675776036 0.078029447 0.674605127
0.758567745 0.928020743 0.090390632
0.258068349 0.428876966 0.590162738
0.593486243 0.431006121 0.254014208
0.176566775 0.577983088 0.173927712
0.802385438 0.625764557 0.46243923

0.467537513 0.622437494 0.799238518
0.884860288 0.475724817 0.878341541
0.967613341 0.122788849 0.299453033
0.384837579 0.975859572 0.378739061
0.302558613 0.124815638 0.962913546

EA26 4.67 3.52 4.83 71.2 90.8 110
1.0
4.6703661957 0 0
-1.1870902094 3.3132673178 0
-0.067553410144 1.6301936195 4.5434742407
4 12
Direct
0.06631151 0.484600842 0.392383692
0.734538272 0.176765781 0.285372683
0.04919827 0.422695128 0.048322632
0.719811313 0.53078104 0.938763718
0.763416615 0.300304773 0.502853131
0.697891617 0.862431033 0.907307285
0.204106519 0.294609148 0.546956804
0.386055388 0.990944652 0.707194788
0.536936789 0.344541211 0.205828252
0.081650707 0.806386215 0.424022919
0.015130144 0.71163426 0.83198452
0.153915092 0.214480833 0.968299517
0.632838807 0.805173774 0.354714734
0.588626662 0.35789024 0.7794499
0.389507911 0.830263871 0.639883713
0.245827839 0.700052015 0.128058258

EA27 4.58 3.55 4.78 78.9 110 104
1.0
4.5824123369 0 0
-0.87816025904 3.4352556815 0
-1.6329177623 0.52948927273 4.4591460111
4 12
Direct
0.95337335 0.635251194 0.585305905
0.663031346 0.843490299 0.303703797
0.651742079 0.689263634 0.678770925
0.032152438 0.828890268 0.918535123
0.49889499 0.411513077 0.774196728
0.051512106 0.571908281 0.114071793
0.265972551 0.239553442 0.298542193
0.679820328 0.167295524 0.180710173
0.497528342 0.913157302 0.476740998
0.98472445 0.330328288 0.567365378
0.972396179 0.861682373 0.355305608
0.75131908 0.955590712 0.849507306
0.519249843 0.6002263 0.146479696
0.283425622 0.263208845 0.45482378
0.210548329 0.820149766 0.743126012
0.179235425 0.141734867 0.9788543

EA28 4.94 3.49 4.5 76.8 72.7 79.4
1.0
4.935188779 0 0
0.64086741244 3.4331761781 0
1.3383091517 0.79516795092 4.2214777851
4 12
Direct
0.489912438 0.832503742 0.526833718
0.724208502 0.913989613 0.177264478
0.836037598 0.588780267 0.490473318
0.393284066 0.775934983 0.203645521
0.935152406 0.77847575 0.605362981
0.792862814 0.228304576 0.075195954

0.367531362 0.466250616 0.161809125
0.397110889 0.087061093 0.669664762
0.085660497 0.380743699 0.859257006
0.230129533 0.04042613 0.133760684
0.935052023 0.65175895 0.169542671
0.932464133 0.252070418 0.510049792
0.182122709 0.483598371 0.905410684
0.580562078 0.507971284 0.68472711
0.656490886 0.790594497 0.975989975
0.291599919 0.667031257 0.518377641

EA29 3.58 3.49 6.17 104 74.4 88.8
1.0
3.5779812224 0 0
0.073109392323 3.4923972541 0
1.6604117909 -1.4804139315 5.7540193284
4 12
Direct
0.490982767 0.330529216 0.77736416
0.387790986 0.235394514 0.264039596
0.05587186 0.573628849 0.912442512
0.158315433 0.669183018 0.425733588
0.700368218 0.13861272 0.267052604
0.900039534 0.571574109 0.103329628
0.626600231 0.146407687 0.878295923
0.155635544 0.330064991 0.471634088
0.645883827 0.333458874 0.586486476
0.390863125 0.574600432 0.218248756
0.147771827 0.213650144 0.793299955
0.331093801 0.864666521 0.540122118
0.399128725 0.690062384 0.897295849
0.921092328 0.75718789 0.810884727
0.845726142 0.765617925 0.422586081
0.214730618 0.040281388 0.149675056

EA30 4.05 2.95 6.18 94.4 109 88.6
1.0
4.0521067437 0 0
0.073979824524 2.9532803022 0
-2.0054078896 -0.42697654782 5.8316179695
4 12
Direct
0.407569972 0.212071929 0.274476745
0.652814414 0.694527845 0.22281473
0.096396118 0.287530777 0.728182864
0.850794935 0.805149839 0.779251461
0.135954184 0.35702388 0.170368241
0.649410111 0.478607372 0.390857329
0.927500099 0.85222862 0.326295076
0.410806889 0.94316167 0.105852846
0.367612621 0.142211668 0.832618728
0.855466361 0.020325104 0.611327951
0.576270845 0.647424576 0.675644308
0.091991533 0.556318666 0.896592195
0.840613162 0.999676455 0.949173389
0.118494512 0.418920235 0.559138268
0.662166386 0.50067666 0.052494806
0.386580404 0.081078405 0.44390316

EA31 3.51 3.49 5.78 91 89.8 87.5
1.0
3.5082312799 0 0
0.15512923874 3.4868376195 0
0.022332545211 -0.098608503432 5.7827553658
4 12
Direct
0.998766143 0.15299896 0.676225491

0.356572725 0.468614825 0.018921651
0.001681736 0.654602164 0.179849641
0.361842462 0.96637423 0.519063826
0.99353045 0.481066212 0.981489952
0.892177378 0.474441813 0.665543458
0.467420535 0.149298734 0.031113614
0.358516204 0.1436181 0.718012445
0.854588496 0.947211625 0.805263161
0.842866857 0.471258928 0.313898744
0.442341077 0.640118121 0.540378098
0.498680338 0.6758634 0.890912093
0.527162693 0.150755582 0.38703513
0.357963612 0.606658454 0.226312213
0.917468235 0.978939744 0.158327643
0.006765315 0.019161438 0.467515143

EA32 3.44 3.47 5.92 91.5 89.1 89.5
1.0
3.4363379298 0 0
0.029378717828 3.470141538 0
0.088877762373 -0.1539593024 5.9185113948
4 12
Direct
0.447254028 0.36943253 0.758933927
0.447165076 0.869372251 0.258774496
0.097900287 0.542280529 0.932041627
0.098189468 0.04222573 0.432182478
0.074183153 0.853400358 0.242493144
0.977113885 0.349081092 0.075613513
0.567469404 0.057063259 0.782867165
0.567398105 0.557002852 0.282772088
0.568220975 0.562470188 0.615341
0.977887006 0.354603938 0.408263448
0.074427824 0.354250917 0.742087697
0.977719162 0.84943046 0.576043183
0.470736637 0.55753116 0.948848584
0.977671482 0.854669137 0.908141967
0.471210089 0.058147838 0.448447551
0.567705879 0.062057349 0.11488103

EA33 3.64 3.55 5.59 90 89.9 85.3
1.0
3.6360080636 0 0
0.28992447118 3.5406251026 0
0.011303339268 -0.0024280270705 5.5905368246
4 12
Direct
0.648328312 0.870791276 0.575998732
0.470712497 0.168957862 0.075969879
0.09772801 0.376934153 0.205994747
0.020136635 0.662905188 0.706262952
0.129994068 0.202482295 0.007169138
0.488087259 0.683946522 0.446192034
0.559583242 0.849516915 0.101472542
0.5569342 0.189901322 0.600799906
0.023915395 0.69788389 0.175398688
0.990310664 0.839770584 0.508402389
0.916917932 0.206522108 0.333542237
0.429275083 0.323087273 0.28435925
0.201142849 0.831070381 0.834543024
0.633382188 0.353550126 0.945754432
0.688712773 0.719459667 0.785123253
0.09227352 0.341848301 0.674644521

EA34 3.46 3.44 5.93 89 91.6 89.7
1.0
3.4642016505 0 0

0.016440382572 3.4363617726 0
-0.16771020411 0.10348552931 5.9274466396
4 12
Direct
0.90108682 0.920151758 0.393396113
0.073342528 0.571023185 0.56691068
0.401104887 0.92021633 0.893397495
0.573321684 0.57099877 0.066900918
0.381893005 0.452862149 0.212016318
0.884117687 0.447563121 0.041744136
0.090292159 0.043627512 0.918568309
0.880100425 0.547307713 0.378527765
0.380170249 0.547350269 0.878522692
0.092522959 0.038284165 0.248291167
0.590306565 0.043602454 0.418558945
0.881960358 0.452820532 0.712031343
0.592562857 0.038357238 0.74829006
0.094208422 0.943914198 0.581793668
0.384137752 0.447596602 0.541742253
0.594255548 0.943831148 0.081799225

EA35 3.37 6.96 3.44 89.9 61.4 90
1.0
3.3704415382 0 0
0.0041129170752 6.9636263517 0
1.6488037456 0.005430400034 3.0233603176
4 12
Direct
0.835829631 0.587876192 0.502102666
0.335258229 0.910701235 0.498132025
0.662281076 0.087751546 0.500706881
0.165811452 0.408998937 0.493550913
0.88756225 0.655188384 0.16973569
0.047286167 0.853415141 0.835428775
0.616857276 0.15041857 0.833796025
0.456900373 0.354750426 0.15524895
0.794396257 0.405614333 0.475039861
0.285051574 0.093702778 0.493518535
0.70787459 0.904730071 0.51281627
0.211010125 0.591073954 0.51571115
0.11226194 0.341847181 0.826447408
0.951041963 0.144586067 0.163599429
0.384318264 0.849296909 0.162773409
0.542923434 0.641287568 0.840311445

EA36 3.46 6.85 3.4 89 60.9 90
1.0
3.4646185932 0 0
-0.0034778707605 6.8514379743 0
1.6518795037 0.058828088897 2.973283154
4 12
Direct
0.494735995 0.662190438 0.177722887
0.498004676 0.835290699 0.82683447
0.494569346 0.162381154 0.177678396
0.498064772 0.335116463 0.825984725
0.158014651 0.606106861 0.473782855
0.162963019 0.898799576 0.879885744
0.157726414 0.106374443 0.473664437
0.162909869 0.39836945 0.878822603
0.481068168 0.64788981 0.80760085
0.481335888 0.147720119 0.807391977
0.51166198 0.849571004 0.197004577
0.511521582 0.349799588 0.196128024
0.834832498 0.391172982 0.529949406
0.829666469 0.099056927 0.124862826
0.834736611 0.891325206 0.530720437

0.829818556 0.598709514 0.124614929

EA37 2.96 3.83 6.07 90 90 90
1.0
2.9641632115 0 0
-0.00015987977058 3.8269548424 0
-8.1634350424e-06 -0.00052313466098 6.0745307907
4 12
Direct
0.689368451 0.381098531 0.460564573
0.189366949 0.194627942 0.601172604
0.204826786 0.88110861 0.101178443
0.704823202 0.69463361 0.960569764
0.049703328 0.820030622 0.274304989
0.037395164 0.911869561 0.598846721
0.844541901 0.320096322 0.28742814
0.344520143 0.255699397 0.774308343
0.549692577 0.755668213 0.787437539
0.537386369 0.663846165 0.462933782
0.856660062 0.411841364 0.962909406
0.356836842 0.163851069 0.098914331
0.932483429 0.457036165 0.625470536
0.432517875 0.118651697 0.436282973
0.961719186 0.957051127 0.936289083
0.461600368 0.618661912 0.125408546

EA38 3.46 4.52 4.89 84 104 72.8
1.0
3.4557455125 0 0
1.3347303019 4.3172691875 0
-1.1934144425 0.90424688651 4.6553856312
4 12
Direct
0.242941091 0.552545758 0.423711181
0.825760176 0.913401707 0.238171196
0.749440714 0.27284182 0.975111536
0.038142416 0.831785271 0.6219376
0.53685557 0.461078814 0.07282098
0.172347488 0.311682514 0.417610847
0.968924031 0.63855484 0.171186675
0.055064095 0.059358166 0.167468494
0.56588985 0.18708759 0.786106045
0.675128712 0.002378223 0.448008531
0.037915075 0.334385781 0.92663116
0.120946937 0.532378715 0.653113179
0.589168446 0.537991097 0.442728728
0.276764807 0.960668808 0.708004907
0.496118403 0.909702413 0.103079033
0.84073945 0.779055256 0.775505986

EA39 2.92 3.96 6.09 93.5 90 90
1.0
2.9238563326 0 0
0.00047859158941 3.9642630131 0
-6.2920034505e-05 -0.37178915875 6.0809018273
4 12
Direct
0.720971927 0.198844606 0.566281365
0.220937674 0.959240424 0.657740082
0.024159741 0.679846359 0.181469737
0.524289448 0.478316361 0.042482125
0.677555397 0.203468893 0.039565003
0.353483265 0.535985124 0.875235147
0.974233267 0.965537927 0.488680314
0.876956202 0.453981685 0.640245364
0.37703385 0.704095172 0.583831468
0.853919352 0.621971683 0.348827186

0.281636905 0.428134108 0.210263484
0.177315906 0.954685265 0.184441413
0.474213042 0.192449451 0.735299627
0.56624628 0.210793184 0.388414495
0.781190995 0.730369987 0.013753991
0.066387935 0.947493318 0.835667588

EA40 3.48 4.47 4.98 108 99.6 76.5
1.0
3.4768407134 0 0
1.0460111039 4.34559712 0
-0.82897811526 -1.3623281557 4.7204773564
4 12
Direct
0.712889463 0.077430422 0.942627748
0.842520117 0.721872196 0.716014484
0.040744094 0.774586982 0.067447358
0.867807509 0.053053 0.61870088
0.283331525 0.398459329 0.295203356
0.595882198 0.093434016 0.454516755
0.383587926 0.158286271 0.98796908
0.94622805 0.105079532 0.163784938
0.605795204 0.564986587 0.605186275
0.828935822 0.2862957 0.878109685
0.192140084 0.349451481 0.395809252
0.164617635 0.575784688 0.819115066
0.044087435 0.743939316 0.541044144
0.369313394 0.772607526 0.176312971
0.158147522 0.123474095 0.588058473
0.849887815 0.651783103 0.158855215

EA41 4.06 2.92 5.95 89.9 90 90
1.0
4.060097033 0 0
0.00014854810851 2.9244612894 0
-0.0048160442174 0.0061522546095 5.9509079301
4 12
Direct
0.62620674 0.936802144 0.044943942
0.868734251 0.436909993 0.954281693
0.126710441 0.933834347 0.453905006
0.368879574 0.433624771 0.545282956
0.644323284 0.184238273 0.870634736
0.12089208 0.280866351 0.012330342
0.850506197 0.683478375 0.128574579
0.373960514 0.781270406 0.986851224
0.874430819 0.777159692 0.510996691
0.351432918 0.681056024 0.370814982
0.621386033 0.277739256 0.488194401
0.144218146 0.180430037 0.628405997
0.398047145 0.600347989 0.722522505
0.097469398 0.101608609 0.276729725
0.898876577 0.604070519 0.77707218
0.596425398 0.102979603 0.222249788

EA42 3.53 4.89 4.7 87.5 111 111
1.0
3.5257037202 0 0
-1.7370028192 4.5697374265 0
-1.7169813179 -0.43186900667 4.3545179767
4 12
Direct
0.691391127 0.398917275 0.770146908
0.748763771 0.853727844 0.420320085
0.062107733 0.743816454 0.752936958
0.79035817 0.507138524 0.438144161
0.519773184 0.591312272 0.235923545

0.231733901 0.158994826 0.084112288
0.402257429 0.095819546 0.110904003
0.441805002 0.820363534 0.852971328
0.485555105 0.295173081 0.471392489
0.908533154 0.661825513 0.95677379
0.423024596 0.880000726 0.401412938
0.850450027 0.233222563 0.901021196
0.360371339 0.373827358 0.790147096
0.938738256 0.958221875 0.719714629
0.992844615 0.428757056 0.335562624
0.946512047 0.013371452 0.287364431

EA43 2.96 3.83 6.08 90 90 90
1.0
2.9638660399 0 0
-5.1267470944e-05 3.8269393477 0
-5.0450309196e-05 -0.00080558395867 6.075775042
4 12
Direct
0.700939852 0.253615856 0.454232156
0.200926619 0.067029003 0.594750257
0.216815037 0.753698497 0.094770727
0.716810234 0.567231323 0.954191693
0.368836627 0.036448597 0.092493655
0.561500265 0.628201831 0.781123732
0.85618445 0.192829359 0.281112784
0.868906255 0.284518108 0.956502436
0.048871326 0.784300125 0.592273749
0.548817352 0.536314884 0.456738359
0.061588572 0.692595334 0.267835083
0.356144628 0.12794159 0.767868322
0.944018138 0.329419654 0.619154534
0.444079801 0.991194412 0.429857923
0.973578921 0.829743305 0.929956978
0.473690035 0.491242365 0.119029562

EA44 3.59 3.73 5.37 93.3 95.4 85.5
1.0
3.5946363171 0 0
0.29389902383 3.7157934657 0
-0.50809554819 -0.26761698657 5.3392748911
4 12
Direct
0.399316722 0.656860028 0.094257219
0.899528058 0.157490705 0.594429634
0.130477311 0.766950211 0.515761162
0.630398038 0.266230555 0.0159239
0.48938002 0.081814394 0.855575568
0.989004008 0.582598211 0.355558151
0.107689769 0.73772624 0.992548378
0.137192438 0.083054407 0.430453233
0.422333722 0.685583945 0.616989814
0.53982058 0.841731101 0.254541707
0.637497893 0.58230743 0.930608625
0.607705823 0.238410164 0.493031248
0.922094476 0.184775963 0.117343989
0.893413304 0.841635823 0.680142313
0.040390647 0.342285098 0.754588816
0.392726145 0.340979762 0.179924699

EA45 3 3.87 6.16 78.5 90 90
1.0
2.998447418 0 0
0.0010817244392 3.8669422843 0
0.0027303442333 1.2288496126 6.0331962284
4 12
Direct

0.764672549 0.958699604 0.278290859
0.264897037 0.740148104 0.185497805
0.75756637 0.456989711 0.779576098
0.257793271 0.241929916 0.684256844
0.930930828 0.231790649 0.206996717
0.507240671 0.502535621 0.608820051
0.430974081 0.466830357 0.256493872
0.604406089 0.959477778 0.451313962
0.097637158 0.246737633 0.510712623
0.514378163 0.998878116 0.107608919
0.014349813 0.699976393 0.356199496
0.006960866 0.196414575 0.854883383
0.4225861 0.966963263 0.751872852
0.923380578 0.731387506 0.7117898
0.104956953 0.739904487 0.012441355
0.598443469 0.452806185 0.953368907

EA46 3.67 3.59 5.22 94.9 90 90
1.0
3.6744717398 0 0
-0.00026233192921 3.5871113235 0
0.00033831799104 -0.44684227743 5.201413751
4 12
Direct
0.099686198 0.126762423 0.113676494
0.651373208 0.62670661 0.613677626
0.40440271 0.462043169 0.307595047
0.346721994 0.961999213 0.807628541
0.141739353 0.617012347 0.387579554
0.60208092 0.317285069 0.478008746
0.363970213 0.103072122 0.271982463
0.149013208 0.81729057 0.978080961
0.016091709 0.387935859 0.008009716
0.171596359 0.08401841 0.647173653
0.579622481 0.584003629 0.14713907
0.60941147 0.116886194 0.887652106
0.858226654 0.988182854 0.217430547
0.735085849 0.887920721 0.508071502
0.892722814 0.488027236 0.71744638
0.386980838 0.603020844 0.771935853

EA47 3.54 4.4 5.04 81.2 89.9 66.3
1.0
3.5378776918 0 0
1.7661098221 4.0285083667 0
0.0076698698203 0.83777487817 4.9660033486
4 12
Direct
0.423983418 0.741360204 0.058833961
0.280599769 0.027578223 0.259270776
0.663441283 0.630048146 0.381754024
0.410877243 0.138465092 0.936399613
0.154610683 0.669343918 0.00706721
0.360306133 0.271079129 0.171871162
0.939080869 0.099631795 0.311089693
0.121241738 0.30226918 0.784428953
0.53640824 0.465863489 0.533095175
0.042050718 0.790354441 0.669746495
0.707295544 0.190926087 0.900885366
0.012045943 0.577532789 0.417912249
0.525138511 0.950096907 0.458724113
0.746555114 0.497952766 0.146331127
0.59241951 0.818879169 0.859403903
0.950804397 0.976679257 0.648702163

EA48 4.03 6.11 2.95 89.5 90.4 74.8
1.0

4.0254120359 0 0
1.6025909773 5.9000656418 0
-0.019109418022 0.030914400026 2.9485507369
4 12
Direct
0.649741492 0.726254194 0.497356675
0.346558895 0.275826173 0.467610539
0.356485298 0.775096422 0.993673384
0.639782157 0.226887276 0.971175436
0.819237581 0.557704351 0.659603906
0.177195068 0.44441571 0.305381267
0.178089205 0.668640149 0.845707958
0.818469418 0.333162995 0.118997738
0.183572668 0.943147961 0.144696143
0.812437613 0.05888835 0.819664574
0.829685512 0.832009746 0.349365757
0.166407158 0.170211583 0.6155676
0.486500489 0.391292614 0.722143515
0.49690006 0.890657118 0.738547693
0.499396273 0.111395688 0.226465908
0.509910634 0.610741349 0.242877443

EA49 4.87 4.78 3.49 110 69.1 104
1.0
4.8664064244 0 0
-1.1589680205 4.634685948 0
1.2421369036 -0.91219664109 3.1265234308
4 12
Direct
0.365779345 0.434151248 0.036332802
0.366950064 0.781497704 0.030813551
0.867389051 0.281048508 0.529604639
0.86511925 0.933825774 0.536587372
0.286951031 0.229225235 0.780173621
0.443978006 0.398502581 0.295109143
0.942163952 0.899079237 0.797068239
0.289177528 0.81618611 0.770974737
0.445920537 0.986834457 0.286329586
0.071927622 0.109425408 0.342771111
0.571741092 0.610114817 0.842497364
0.786180881 0.728381352 0.281158532
0.79125673 0.316212513 0.268592771
0.660264405 0.105312851 0.722386316
0.945723799 0.485974459 0.786169039
0.160948248 0.605826918 0.225066559

EA50 3.48 3.44 5.92 89.5 88.6 91
1.0
3.4767205342 0 0
-0.06039481366 3.435424844 0
0.14435163653 0.05932637236 5.9162157076
4 12
Direct
0.421418671 0.117595835 0.189522252
0.60031719 0.465591698 0.017772396
0.100814234 0.466916734 0.517637328
0.921916954 0.118907707 0.689408016
0.614980918 0.989991286 0.330302923
0.916446204 0.576912991 0.040692412
0.105355709 0.005007198 0.167724447
0.417778161 0.490489541 0.209403398
0.409272037 0.593901667 0.876030218
0.920973401 0.491874588 0.709732188
0.104453287 0.094027499 0.497786489
0.112976492 0.990615043 0.831154588
0.416875758 0.579519623 0.539445159
0.907204973 0.594527649 0.37687812

0.601288489 0.092611104 0.997423738
0.605774912 0.007611125 0.666484844

EA51 4.46 3.47 4.98 80.8 72.5 77.1
1.0
4.4619692419 0 0
0.77496157219 3.3806383011 0
1.5000735682 0.47178331331 4.7248088566
4 12
Direct
0.939967799 0.27739297 0.431038998
0.640871935 0.602606077 0.303562905
0.919228689 0.436509202 0.753279223
0.585798427 0.414486435 0.656437913
0.215063338 0.766633908 0.978687535
0.971095005 0.506204012 0.208079849
0.642646722 0.928428717 0.190750769
0.15206278 0.391099645 0.494713042
0.515106822 0.408035637 0.215703017
0.995569604 0.725243917 0.782625273
0.444045225 0.734782914 0.549643932
0.426105585 0.179834743 0.768843973
0.611890796 0.618220286 0.829861853
0.014274069 0.947089775 0.388492638
0.955327159 0.165009021 0.917955443
0.266554675 0.854727488 0.080194003

EA52 3.44 6.47 3.44 105 89.4 105
1.0
3.4447352525 0 0
-1.6883807764 6.2456044814 0
0.035542634104 -0.92368365568 3.3152128109
4 12
Direct
0.186844661 0.512771612 0.45988891
0.098068628 0.688273785 0.216548467
0.520786847 0.188555312 0.795211612
0.76416323 0.012479464 0.88126745
0.511420223 0.340554037 0.064319365
0.224916404 0.513072001 0.08910393
0.06002603 0.687923639 0.58737421
0.364765971 0.841268541 0.207715673
0.500393419 0.546780203 0.625893504
0.929023155 0.045113453 0.194982856
0.355930684 0.15592248 0.48149443
0.920180212 0.359782439 0.468740329
0.393703436 0.012430482 0.918808503
0.773531525 0.860486096 0.612147493
0.784481708 0.654251779 0.050562857
0.891248249 0.188600691 0.757682704

EA53 3.66 4.69 4.69 80.6 112 112
1.0
3.6649360475 0 0
-1.7897615052 4.3368720787 0
-1.7899485508 0.092882696017 4.335211264
4 12
Direct
0.028565764 0.67332348 0.517898753
0.026978943 0.171358739 0.016227101
0.697010105 0.899408407 0.243878755
0.744911573 0.446262094 0.790946963
0.705614652 0.627711278 0.27667256
0.571563028 0.544640958 0.893590649
0.339037454 0.889225719 0.572641176
0.078414745 0.457907493 0.465808275
0.990128467 0.415127391 0.061674212

0.494219588 0.263124324 0.60708475
0.335985338 0.22359134 0.233602911
0.327134383 0.794740289 0.142860069
0.077262305 0.116750796 0.802256763
0.97640193 0.720278401 0.755007112
0.80075502 0.082655222 0.426467851
0.694601471 0.93657707 0.969227468

EA54 3.52 3.35 4.82 90.6 86.5 90.2
1.0
3.5186607619 0 0
-0.010802644145 3.3520496973 0
0.29413563277 -0.049927616824 4.8077464233
4 12
Direct
0.897724019 0.58258402 0.800738505
0.472989414 0.078256794 0.359839785
0.024719491 0.426037342 0.120562546
0.600175905 0.921697659 0.679704552
0.070776714 0.084380668 0.111699138
0.936543624 0.95096886 0.800162205
0.79304356 0.052148254 0.468544278
0.241492367 0.882927595 0.486915117
0.081064961 0.403812919 0.63380913
0.932010218 0.587433322 0.324995994
0.565770617 0.916841583 0.155409204
0.41671494 0.100483202 0.846548043
0.561215098 0.553401866 0.680228544
0.427116077 0.419951266 0.368690705
0.256085047 0.621468891 0.993358211
0.704708975 0.451957323 0.011817561

EA55 3.15 2.9 6.75 85 78.1 71.8
1.0
3.1548598716 0 0
0.90465722728 2.7559961752 0
1.3881469744 0.16633005089 6.6027814367
4 12
Direct
0.583852608 0.021022238 0.073350197
0.721251124 0.502437031 0.918368689
0.416761894 0.479192847 0.428726764
0.282802167 0.996514025 0.583871714
0.944771116 0.912261663 0.642844491
0.759532026 0.249822931 0.334993314
0.061965536 0.582414509 0.861404142
0.240970792 0.250248213 0.166877731
0.190050293 0.729211571 0.321529231
0.396062182 0.093484446 0.721814793
0.626379263 0.65874042 0.528785292
0.09303618 0.300991489 0.466997263
0.809242583 0.770263545 0.180841676
0.611264331 0.407901631 0.778952856
0.37829215 0.844136438 0.970637615
0.908232423 0.196908061 0.035951696

EA56 3.5 3.31 4.96 90 86.5 90
1.0
3.4992600362 0 0
3.8923316104e-05 3.3112653894 0
0.30408883752 -4.7637723667e-05 4.9537960736
4 12
Direct
0.358195877 0.25498036 0.806324967
0.358331234 0.940636013 0.306316053
0.772135788 0.754979246 0.195771961
0.771993964 0.440634565 0.695763506

0.448217582 0.762550995 0.086711864
0.296076841 0.278367181 0.275207881
0.16294729 0.738541896 0.448021591
0.295579019 0.917280877 0.775470985
0.162962279 0.45758114 0.947794374
0.448208215 0.432448263 0.586527234
0.967354597 0.957526729 0.054263385
0.682154491 0.932532198 0.415524683
0.967423215 0.238516289 0.554104986
0.834787311 0.417302597 0.226677227
0.682150842 0.262634135 0.915390428
0.834242225 0.778374625 0.726815566

EA57 3.26 3.18 5.58 90.1 88.8 90
1.0
3.2550306182 0 0
-0.0012941792142 3.1842184211 0
0.11320999885 -0.013258495292 5.576592302
4 12
Direct
0.592035215 0.071106144 0.906776449
0.907526281 0.422609235 0.591966142
0.407634783 0.422564282 0.092002079
0.09186167 0.071101104 0.406783282
0.421916729 0.948651291 0.4321678
0.077602023 0.545112742 0.066663657
0.889020209 0.95090426 0.253643077
0.610433334 0.542835193 0.245149686
0.110628426 0.542718815 0.745014829
0.922117691 0.948629105 0.932081212
0.389160908 0.951055718 0.75359729
0.577547968 0.545291317 0.566557714
0.892621339 0.02481597 0.603796511
0.606891385 0.468912724 0.894996132
0.393029241 0.024752565 0.103865918
0.106625745 0.46888103 0.394914216

EA58 4.54 3.27 4.53 69.3 107 79.5
1.0
4.543782844 0 0
0.59769558516 3.2104795962 0
-1.3504259923 1.8772539348 3.8904193123
4 12
Direct
0.30379165 0.704397156 0.591929581
0.300917466 0.687322754 0.204864708
0.695743104 0.290042316 0.411149016
0.698833261 0.307187052 0.798076504
0.66275755 0.976422766 0.965555832
0.813755451 0.509132631 0.249953545
0.920082855 0.343230797 0.991970996
0.417532907 0.284660769 0.242605139
0.33754783 0.017680171 0.037652589
0.185739589 0.48530689 0.753075864
0.07933434 0.651866351 0.010981996
0.581956237 0.70967969 0.760542483
0.775383815 0.914317734 0.444667774
0.900194054 0.310867493 0.665253373
0.224135416 0.080307595 0.558087154
0.099376031 0.684875621 0.337162358

EA59 4.49 3.19 4.52 70.3 90.6 70.4
1.0
4.4850230246 0 0
1.0663523803 3.0022040894 0
-0.0439444827 1.6328719391 4.2180093481
4 12

Direct
0.073738043 0.666833948 0.31259135
0.458546437 0.263067401 0.339474622
0.020976453 0.655603217 0.710189272
0.43281657 0.347016934 0.927558035
0.515087623 0.98944323 0.16830787
0.952248574 0.032877251 0.112980754
0.038307183 0.372474211 0.231584789
0.466070143 0.925693806 0.552237439
0.336245497 0.66365221 0.392015099
0.687883361 0.349540146 0.372498184
0.89007296 0.562947794 0.506880843
0.442267204 0.702221759 0.934818931
0.196620957 0.240977103 0.877805443
0.628892566 0.18695137 0.793161208
0.032329408 0.019129538 0.678820885
0.810192115 0.600515016 0.843441119

EA60 3.26 3.18 5.58 90.1 88.8 90
1.0
3.2550306182 0 0
-0.0012941792142 3.1842184211 0
0.11320999885 -0.013258495292 5.576592302
4 12
Direct
0.592035215 0.071106144 0.906776449
0.907526281 0.422609235 0.591966142
0.407634783 0.422564282 0.092002079
0.09186167 0.071101104 0.406783282
0.421916729 0.948651291 0.4321678
0.077602023 0.545112742 0.066663657
0.889020209 0.95090426 0.253643077
0.610433334 0.542835193 0.245149686
0.110628426 0.542718815 0.745014829
0.922117691 0.948629105 0.932081212
0.389160908 0.951055718 0.75359729
0.577547968 0.545291317 0.566557714
0.892621339 0.02481597 0.603796511
0.606891385 0.468912724 0.894996132
0.393029241 0.024752565 0.103865918
0.106625745 0.46888103 0.394914216

EA61 3.27 3.14 5.65 90 90.5 90
1.0
3.2683361261 0 0
0.00013742057434 3.1351048137 0
-0.048440610512 0.00064480231964 5.6521848337
4 12
Direct
0.659327898 0.980820587 0.658604411
0.844887914 0.632688937 0.8445274
0.345113494 0.480494748 0.344511194
0.159447289 0.132289559 0.15873583
0.143748532 0.60200566 0.497597419
0.854883658 0.036429372 0.849985229
0.827655358 0.012524915 0.182359542
0.676874963 0.600075769 0.320621136
0.176570002 0.512654402 0.820840858
0.150713168 0.536117982 0.152707448
0.327821857 0.101092176 0.68293944
0.650645915 0.576834922 0.652795941
0.860587536 0.101810412 0.505346898
0.361626908 0.010711978 0.006039762
0.643340724 0.511957139 0.99764903
0.352867848 0.076631075 0.350788841

EA62 4.47 3.35 4.47 79.4 65.3 68.2

1.0
4.4727131338 0 0
1.2445922924 3.1069087019 0
1.8679504001 0.13623140255 4.0560769694
4 12
Direct
0.821829409 0.481676693 0.616295411
0.204469657 0.078221614 0.619824663
0.05923451 0.833248396 0.004605491
0.676464441 0.236927133 0.001145783
0.818519729 0.055903877 0.224826961
0.166563792 0.803905782 0.211967497
0.468772842 0.068224948 0.077636452
0.57568701 0.545067339 0.150728598
0.062234491 0.258784837 0.395990345
0.573497404 0.347638274 0.745937332
0.307438283 0.967567973 0.875077226
0.812018822 0.817399674 0.646241664
0.305503404 0.770198628 0.470071488
0.068958194 0.497653428 0.974428302
0.71463393 0.510643929 0.408882538
0.411897062 0.247305026 0.543357463

EA63 3.3 3.08 5.63 90.5 90.1 90.5
1.0
3.303179444 0 0
-0.025158055761 3.0827641733 0
-0.0097404041209 -0.044557102043 5.6339147518
4 12
Direct
0.536502538 0.222242502 0.291298678
0.083629467 0.301668856 0.799309513
0.725033557 0.886421422 0.48302618
0.186972546 0.805793198 0.967441227
0.735486335 0.299019553 0.477124942
0.757081304 0.157220421 0.783735801
0.212875338 0.358948798 0.307668784
0.746304865 0.319328908 0.136602557
0.228121437 0.333441238 0.615282567
0.375356784 0.141038002 0.903453923
0.514082372 0.676365297 0.98638384
0.892001623 0.629495583 0.865528214
0.052619628 0.768407994 0.458589001
0.522065227 0.816103029 0.292097219
0.038201559 0.837169611 0.150668394
0.524757494 0.75344949 0.634877473

EA64 2.83 6.55 3.05 90 90 90
1.0
2.8268327767 0 0
0.00031244072274 6.5529393081 0
-0.0001291045517 -0.00029058978163 3.0487192912
4 12
Direct
0.679317654 0.434324944 0.23379506
0.179428191 0.565557331 0.046114296
0.179313559 0.434241374 0.546112638
0.679425367 0.565639694 0.733817334
0.59911467 0.92624293 0.061586247
0.596805404 0.073735396 0.562054269
0.253291998 0.869858018 0.56562967
0.096263187 0.073175783 0.214328077
0.685476119 0.736580982 0.642595305
0.686336698 0.263462589 0.141913617
0.185780666 0.736456619 0.13759322
0.185894805 0.263291939 0.637367081
0.255790785 0.130511673 0.067934231

0.098022479 0.925989336 0.717990555
0.754133142 0.130248406 0.711943556
0.753180453 0.869593514 0.214189592

EA65 3.34 3.34 5.1 90 90 90
1.0
3.3433439336 0 0
0.0016663508177 3.3440112847 0
7.3189159129e-05 -0.00026505194454 5.0989948271
4 12
Direct
0.51635966 0.22758053 0.54374986
0.489162597 0.765386466 0.043735655
0.234202723 0.483301037 0.293843507
0.771210634 0.509769044 0.793881763
0.830397403 0.217126917 0.910289099
0.17495218 0.775823701 0.410423965
0.256715612 0.466822061 0.052579863
0.74827528 0.526565308 0.552666387
0.532470854 0.250202677 0.302379172
0.47247113 0.742259741 0.802404945
0.223779452 0.167920591 0.66023811
0.781839399 0.824882193 0.160117761
0.272706227 0.028588409 0.007923512
0.733136808 0.964591016 0.507941772
0.034678687 0.725718153 0.758482125
0.970610159 0.267513752 0.258455633

EA66 4.47 3.36 4.42 77.2 65.6 68.9
1.0
3.2237007119 3.0961697319 0
-1.2991194836 3.0961697319 0
1.0733029901 1.5160790685 4.011707423
4 12
Direct
0.273581942 0.823170626 0.342886589
0.420965315 0.633299522 0.65355662
0.031518367 0.008748724 0.725486376
0.662862856 0.44731053 0.270742997
0.362043731 0.341625932 0.841358749
0.08693021 0.688719897 0.30104774
0.608527857 0.766902382 0.694638233
0.017311936 0.370506578 0.571101671
0.672510304 0.097738027 0.212721356
0.678004613 0.085015928 0.424157854
0.762619346 0.963010796 0.829342473
0.563547189 0.512808169 0.047961782
0.333724701 0.11388492 0.154248383
0.132455693 0.943444132 0.947195832
0.931818424 0.492616322 0.168001365
0.020335497 0.358857807 0.783547059

EA67 2.89 3.51 5.71 85.1 90 90
1.0
2.8940374169 0 0
-0.0024705017211 3.5054398954 0
0.0037007838017 0.48588576475 5.6866549685
4 12
Direct
0.826391368 0.178731439 0.975470791
0.32657773 0.027354523 0.826220473
0.310548415 0.526498139 0.326431391
0.810177654 0.680363713 0.474979707
0.079836608 0.89313409 0.993136692
0.477318501 0.724338838 0.819037232
0.056353956 0.81154792 0.307020422
0.169338367 0.124070936 0.645628

0.554277766 0.395873506 0.495184691
0.66863535 0.082146342 0.156050067
0.65979897 0.983783049 0.480372317
0.979050349 0.481034641 0.983281481
0.15835032 0.223554384 0.321064692
0.966879493 0.586000328 0.656018202
0.469141203 0.619944416 0.14553358
0.581638012 0.313027049 0.808316075

EA68 3.27 3.29 5.25 89.8 90.5 91.5
1.0
3.2749391472 0 0
-0.08539692714 3.2927118859 0
-0.050184956206 0.013023970206 5.2537349466
4 12
Direct
0.50800877 0.676896009 0.332723844
0.228572943 0.957352723 0.091212773
0.231649777 0.425332121 0.580768396
0.984603781 0.675448591 0.841417098
0.955947976 0.11260276 0.182072241
0.499547254 0.032835041 0.261275952
0.716432323 0.836185747 0.485821764
0.696186206 0.463680712 0.208550051
0.140225288 0.556708813 0.364447679
0.258029852 0.767721505 0.68707401
0.95122431 0.21481072 0.535195937
0.20189261 0.585482205 0.031732558
0.772776321 0.94604206 0.810264843
0.512814475 0.237799299 0.631154994
0.787545264 0.386559224 0.879140836
0.416141008 0.168076617 0.956610521

EA69 4.5 3.28 4.44 70.9 86.8 110
1.0
4.5046012212 0 0
-1.1328621771 3.0823935079 0
0.24905090579 1.6444204817 4.1219638676
4 12
Direct
0.213474728 0.235569677 0.134301053
0.819598296 0.261451431 0.154003595
0.842764146 0.660126911 0.767341101
0.442245739 0.649419175 0.747635805
0.148691209 0.843806438 0.260452902
0.79644311 0.659247357 0.044568412
0.401588616 0.396971264 0.274063798
0.639361466 0.908773185 0.156476362
0.332076466 0.673248051 0.51569265
0.071129136 0.416393209 0.256290732
0.228811429 0.242287218 0.853811294
0.926209593 0.409129913 0.686816137
0.70917236 0.287323217 0.390044598
0.034909535 0.034741781 0.644108935
0.458006595 0.968982545 0.808924737
0.639072396 0.560258245 0.59718546

EA70 4.47 3.28 4.52 69.1 92.2 69.9
1.0
4.4694740928 0 0
1.12767578 3.0852118258 0
-0.17157960205 1.7828550175 4.1539796646
4 12
Direct
0.655552776 0.610701122 0.028693178
0.673432694 0.641804778 0.418879129
0.26056661 0.016468485 0.800381351

```
0.283815574 0.040627299 0.399501071
0.725114355 0.856037829 0.142705179
0.383989546 0.594811881 0.004057631
0.321050852 0.337650048 0.778949212
0.031555099 0.034546266 0.914763014
0.558636115 0.033304443 0.455186354
0.802652407 0.212779135 0.130718728
0.909876026 0.669842236 0.516301233
0.676285306 0.285396514 0.601184304
0.156666372 0.421483104 0.210816292
0.798775983 0.751890443 0.836326982
0.216163677 0.785561289 0.313241645
0.118022392 0.924096216 0.60447097

EA71 4.94 3.36 3.44 90 90 86.3
1.0
4.9368410527 0 0
0.21932526848 3.3518276191 0
-0.0002305691902 -0.00021546515031 3.44036923
4 12
Direct
0.941298217 0.365745177 0.271592194
0.74805451 0.700809649 0.964591704
0.44137322 0.865667804 0.7202334
0.248129087 0.200885107 0.027345274
0.157315872 0.352298239 0.749052514
0.919308263 0.842343818 0.793451345
0.544577838 0.143646159 0.611542153
0.275950978 0.823634052 0.994440608
0.776476999 0.323477709 0.996554919
0.044611905 0.643582776 0.380395372
0.419627498 0.341838564 0.198493536
0.657360587 0.851969289 0.243070189
0.584531877 0.543378119 0.736287555
0.832645168 0.212768685 0.522725129
0.084718063 0.04370618 0.255967602
0.332818847 0.712750855 0.468937325

EA72 3.25 5.58 3.18 90.1 90 88.9
1.0
3.2546415661 0 0
0.11063369877 5.5819993546 0
-0.0010683022562 -0.0070802294879 3.180697653
4 12
Direct
0.907724922 0.370280108 0.14985596
0.408095173 0.870352632 0.149333176
0.092847142 0.184546739 0.799647287
0.593021263 0.684696844 0.798796193
0.106592891 0.173670679 0.198157658
0.394848233 0.881764916 0.75083731
0.111195709 0.523025161 0.269932477
0.923165287 0.709887114 0.675887133
0.89293605 0.381015137 0.751453202
0.890394254 0.031352907 0.679241711
0.389098614 0.532032826 0.678726356
0.077967926 0.84470189 0.271858122
0.611202708 0.022973331 0.271082376
0.577944983 0.344922417 0.273468284
0.606084312 0.673222144 0.197146154
0.422637893 0.210490756 0.67647417

EA73 3.03 5.67 3.27 90 90 90
1.0
3.0302126525 0 0
-0.0019382573332 5.667767569 0
-0.00037861960341 -0.0014394056638 3.2707199612
```

4 12
Direct
0.16027496 0.068433604 0.946409327
0.088070108 0.567504012 0.44506766
0.660358471 0.066684231 0.266187928
0.588310163 0.567402379 0.765035655
0.597178163 0.240301848 0.442179636
0.14889997 0.741313609 0.269429725
0.174138928 0.57790066 0.821367027
0.673757119 0.558777541 0.388307031
0.574802181 0.069653982 0.889043393
0.595774213 0.890521303 0.434456933
0.655407731 0.743265831 0.933918742
0.648077636 0.393440799 0.940361023
0.094938566 0.244506275 0.778390633
0.09782173 0.894774908 0.770189082
0.155043911 0.391373668 0.27729331
0.074808782 0.062633661 0.323250369

EA74 3.26 5.76 3.25 106 89.8 74.5
1.0
3.26425767 0 0
1.5361854493 5.553613605 0
0.010532761031 -0.90947912341 3.1236806953
4 12
Direct
0.565905898 0.741863835 0.471164326
0.800293264 0.233650507 0.725757249
0.643039207 0.994598242 0.888021744
0.427008105 0.485744711 0.598322045
0.937347862 0.342415955 0.02210366
0.242453879 0.438579601 0.297204735
0.856914039 0.70819696 0.241308297
0.052736263 0.087939906 0.460687832
0.257551764 0.768725218 0.290056672
0.42856551 0.06690619 0.208364025
0.194280119 0.538343358 0.898229465
0.726184001 0.296281488 0.391245348
0.983100709 0.891319131 0.950215157
0.455717426 0.195731345 0.798143888
0.694273808 0.609096162 0.713925555
0.330081349 0.926328746 0.734838608

EA75 3.28 4.59 4.42 91.7 69 110
1.0
3.2767515128 0 0
-1.6048892751 4.3003039853 0
1.5860320706 0.45299141297 4.0969568301
4 12
Direct
0.728249685 0.93687841 0.456714391
0.108562836 0.296631206 0.468682948
0.051208774 0.826933593 0.110918516
0.010918725 0.187260859 0.124561089
0.872846618 0.716902413 0.420431261
0.889261533 0.025435298 0.670627682
0.846604277 0.658344086 0.969935887
0.328525966 0.412984231 0.162730532
0.294466718 0.096307533 0.912943276
0.786948726 0.262729717 0.01894528
0.942325529 0.467397595 0.613305113
0.462082243 0.321522981 0.483663691
0.40249161 0.619180469 0.807536884
0.519869963 0.503963495 0.778007039
0.398517235 0.803377844 0.09514893
0.327978124 0.860896498 0.554994647

```
EA76 3.26 4.44 4.57 103 110 103
1.0
3.255337977 0 0
-0.97399882786 4.3298512296 0
-1.5787203307 -1.4119458437 4.0440076746
4 12
Direct
0.950882916 0.746060991 0.612324058
0.025824328 0.262063666 0.023206607
0.955506702 0.865969266 0.027152847
0.012639145 0.372632374 0.430689218
0.339279953 0.29932611 0.541934967
0.287735154 0.658052068 0.609386782
0.843473924 0.425469517 0.167218745
0.88088767 0.606926666 0.806695361
0.611498701 0.729301724 0.403793213
0.713330334 0.190296783 0.445420455
0.220099914 0.997469665 0.680888061
0.302502707 0.117647429 0.139460588
0.219812473 0.817769368 0.245389886
0.326538056 0.433303976 0.995362052
0.691470882 0.178164009 0.781129757
0.602461842 0.818224227 0.046864182

EA77 3.02 5.69 3.27 90 90 90
1.0
3.0205311673 0 0
0.00024090105382 5.6903784974 0
9.5543866457e-06 -0.00047277992235 3.2741837606
4 12
Direct
0.819101449 0.070116518 0.267859863
0.319190833 0.569989238 0.448158124
0.319087018 0.069893299 0.948213875
0.81916733 0.570042373 0.767818317
0.883507049 0.244519783 0.439027931
0.384154807 0.744457593 0.277370398
0.40353967 0.568664644 0.825016572
0.903614784 0.57068849 0.390940716
0.883946535 0.895854836 0.439334377
0.403513202 0.069877291 0.325109377
0.883279762 0.744292527 0.939490756
0.884015952 0.395621831 0.938848719
0.903449342 0.070127021 0.890984777
0.383510889 0.895486152 0.777036221
0.383431347 0.395795894 0.276288754
0.383657552 0.244155376 0.776686911

EA78 3.22 4.48 4.52 90.2 69.6 70.4
1.0
3.2170280535 0 0
1.505283728 4.2209175408 0
1.5754913531 -0.58010159367 4.1932787511
4 12
Direct
0.446510483 0.538392756 0.228070598
0.497174121 0.444683696 0.834306826
0.178623414 0.93650658 0.194097818
0.205686218 0.835544711 0.79311642
0.497517112 0.994906705 0.192934574
0.807112664 0.518455527 0.236013223
0.285126716 0.399744172 0.429635071
0.393364371 0.319976022 0.086424578
0.848122494 0.913135352 0.750006951
0.031356883 0.7507406 0.370567371
0.481535063 0.892874576 0.580547064
0.984481989 0.064747889 0.008012597
```

0.882855722 0.391483769 0.662830485
0.093168843 0.593664271 0.811214572
0.396199892 0.237079055 0.754433717
0.848069107 0.123287739 0.391855265

EA79 3.09 5.64 3.28 89.5 90.1 90.3
1.0
3.0897345298 0 0
-0.02854674587 5.6433589568 0
-0.0038354328792 0.029844832229 3.2775988098
4 12
Direct
0.062220946 0.072873868 0.428147493
0.451589989 0.568311155 0.455350951
0.416878996 0.0628484 0.791671392
0.954783188 0.566533289 0.765079147
0.550410875 0.542286585 0.825888715
0.948118147 0.896579483 0.287537481
0.499957565 0.39634936 0.275372601
0.018546429 0.738033134 0.944618646
0.471419913 0.081287166 0.408582024
0.523883243 0.746439004 0.291820588
0.541140771 0.880498182 0.898900927
0.536987978 0.237924057 0.932783072
0.034383917 0.388290416 0.924925618
0.046235974 0.596941802 0.395914446
0.935568313 0.254933983 0.320267513
0.006606075 0.056039312 0.810484895

EA80 3.27 4.57 4.47 91.1 111 69.1
1.0
3.2711223138 0 0
1.6304122681 4.2664862571 0
-1.6167422658 0.52563316635 4.1312166859
4 12
Direct
0.201160702 0.881196334 0.399132503
0.248827694 0.239394449 0.437058391
0.277711961 0.799384241 0.047497392
0.907335714 0.155743158 0.085448709
0.969607957 0.801597359 0.491592223
0.490981885 0.956052555 0.620609075
0.095447433 0.636637422 0.902382032
0.505223 0.23376232 0.995288707
0.749460876 0.53642883 0.745455096
0.509291676 0.656483995 0.347313132
0.046260336 0.397113454 0.584671211
0.59574822 0.265829837 0.458271771
0.542789639 0.499476525 0.735200767
0.050743601 0.082931782 0.862801899
0.627117758 0.773466149 0.023739425
0.037092769 0.382852431 0.136654325

EA81 4.57 3.24 4.35 112 89.4 73.3
1.0
4.5665055955 0 0
0.93431013889 3.1069264024 0
0.045291810895 -1.6775077433 4.0087768825
4 12
Direct
0.638185829 0.283569663 0.541790514
0.255866848 0.348577645 0.578208823
0.852630687 0.972990829 0.154242073
0.471216501 0.995119101 0.206865896
0.180125438 0.132450598 0.704946101
0.048783671 0.805431576 0.283861345
0.194945859 0.297844968 0.289800661

0.060058934 0.687378526 0.599992675
0.793995332 0.669753282 0.903381595
0.388350814 0.629933363 0.728363041
0.465889354 0.622450851 0.159141894
0.974687169 0.550975705 0.981692486
0.946814564 0.180263492 0.035652906
0.411439634 0.109875178 0.979522995
0.742183385 0.119922891 0.728322751
0.716168128 0.599010154 0.543731689

EA82 4.32 3.28 4.83 97.1 116 69.1
1.0
4.3193071867 0 0
1.1706693627 3.059796179 0
-2.1365270695 0.17951289671 4.328667378
4 12
Direct
0.829421417 0.671754409 0.284713279
0.59095598 0.138903826 0.432340073
0.456917859 0.176485182 0.74139147
0.919090696 0.844577485 0.997727732
0.990709298 0.320633683 0.381516082
0.43414669 0.941846965 0.503245081
0.39042169 0.315687673 0.193724388
0.723455318 0.23430544 0.971596323
0.90790033 0.843691323 0.541251081
0.080798462 0.776624592 0.281600959
0.740100376 0.363634902 0.599714177
0.257427163 0.057757839 0.783602965
0.351354464 0.55480375 0.729927698
0.543670739 0.693334126 0.096223689
0.917675361 0.541277099 0.846737135
0.161473496 0.951935932 0.0251663

EA83 3.27 5.67 3.03 90 90 90
1.0
3.2705592778 0 0
0.0011670381845 5.6687329923 0
-7.1583052048e-06 0.00065107819989 3.0314630705
4 12
Direct
0.02866854 0.000813963 0.283393949
0.528600568 0.50037078 0.212092102
0.84862094 0.501273134 0.712100987
0.348706222 0.000753941 0.783392816
0.905444661 0.503590402 0.298034684
0.405383927 0.994736512 0.197341539
0.859076017 0.176390262 0.216995148
0.35901475 0.324762802 0.277510505
0.023406732 0.327276934 0.775037602
0.523149759 0.174858791 0.721456075
0.517905119 0.825070065 0.717119524
0.017456395 0.677066339 0.77743853
0.354490374 0.674559161 0.27476559
0.854577238 0.826587071 0.221752381
0.972054602 0.007547432 0.697328985
0.471854125 0.496272221 0.798036488

EA84 4.81 3.52 3.35 89.9 89.3 86.5
1.0
4.8141968381 0 0
0.21237918131 3.5115954279 0
0.038981145949 0.0051704306729 3.3530719904
4 12
Direct
0.875178311 0.969525962 0.989879708
0.433963952 0.545376665 0.493958508

0.195497875 0.096193159 0.146384023
0.754231204 0.672226637 0.650239744
0.56093294 0.313695382 0.688644061
0.442426845 0.498296618 0.152380063
0.229362515 0.637999123 0.655654654
0.70866283 0.153768714 0.168774081
0.085733595 0.776373007 0.120673158
0.874962048 0.00871427 0.621750878
0.068661397 0.328041653 0.951584702
0.186495614 0.142090691 0.488136101
0.400197168 0.003128942 0.98500784
0.543654603 0.865212659 0.518144168
0.753864851 0.633512301 0.018660013
0.921177076 0.488137726 0.471682071

EA85 2.85 5.64 3.57 90 90 90
1.0
2.8477743442 0 0
9.9421497904e-05 5.6433332905 0
-0.00076775141986 0.00014653222888 3.5720066375
4 12
Direct
0.125572958 0.999404892 0.520146766
0.621919366 0.641610013 0.019699395
0.625578726 0.141731759 0.704885833
0.121934537 0.499540683 0.204629738
0.286971774 0.317130699 0.13989963
0.960398454 0.816947639 0.584460429
0.960115883 0.497733106 0.501160219
0.460196264 0.643208431 0.723079759
0.287433094 0.997617789 0.223574475
0.786436128 0.824186551 0.084140021
0.787267271 0.143541343 0.001581614
0.461063837 0.324272267 0.639902014
0.862690396 0.46528176 0.926982459
0.362100112 0.676073748 0.296908206
0.88556522 0.175979852 0.42761253
0.385058178 0.965374577 0.797923137

EA86 3.32 4.46 4.46 83.2 112 112
1.0
3.3238960283 0 0
-1.6511785044 4.1421365847 0
-1.6602861858 -0.096450558793 4.1405109134
4 12
Direct
0.024317053 1.479e-05 0.999161116
0.02078351 0.496525451 0.495607574
0.353986007 0.267803768 0.767614231
0.313894502 0.72777547 0.227555738
0.394512764 0.235804313 0.058413916
0.587410749 0.921538146 0.421347116
0.04466467 0.741018626 0.94500603
0.507665155 0.628390055 0.118800369
0.676113935 0.279776281 0.426440624
0.354155423 0.550725131 0.754175807
0.966065105 0.719510149 0.558186503
0.242152431 0.074019703 0.574020125
0.685397651 0.937351069 0.77589177
0.755194511 0.376793206 0.867303258
0.963804938 0.069134597 0.215591865
0.096013174 0.437241289 0.259257678

EA87 4.49 3.18 4.52 70.3 89.5 110
1.0
4.4860234426 0 0
-1.0647848787 3.0015219953 0

0.038668671095 1.6329882407 4.2172395586
4 12
Direct
0.717466984 0.513072509 0.124588392
0.155742741 0.90382297 0.496233081
0.743980121 0.596067476 0.71271386
0.102600759 0.915100263 0.098421023
0.286302233 0.811219704 0.292960464
0.488731465 0.601489766 0.156390096
0.708918044 0.175050696 0.337738715
0.66116361 0.238830669 0.953920424
0.548105442 0.435516517 0.578177816
0.734786166 0.951957088 0.719222297
0.84087909 0.914090097 0.17733468
0.135831052 0.617979442 0.01893787
0.980338328 0.489490828 0.664332015
0.225524568 0.280391247 0.898475741
0.366472588 0.848096991 0.629751718
0.144764875 0.267663075 0.464810725

EA88 3.26 5.76 3.26 74.8 90.5 105
1.0
3.2584750173 0 0
-1.512864303 5.5569528197 0
-0.025920822375 0.87719455661 3.1364144158
4 12
Direct
0.004449969 0.292318764 0.860143088
0.748335077 0.792355165 0.604708946
0.615308579 0.04491019 0.737088789
0.870600262 0.544640558 0.993621598
0.363912299 0.007398542 0.012427514
0.184022954 0.57974476 0.164513509
0.990371641 0.910924266 0.318601408
0.591020263 0.506149252 0.240663748
0.439634948 0.694287855 0.477239871
0.130682299 0.19417478 0.16946693
0.93520076 0.215225091 0.535962519
0.07245393 0.715469509 0.675256374
0.291641461 0.410138081 0.618838725
0.668110683 0.360184874 0.874263365
0.730860005 0.859637207 0.941846799
0.448986097 0.079564538 0.420739502

EA89 2.96 4.59 4.34 83.8 76.5 100
1.0
2.9627491412 0 0
-0.83282317541 4.5098154993 0
1.0171352092 0.66526736387 4.1693954712
4 12
Direct
0.814071034 0.085425303 0.743664671
0.231782174 0.085622935 0.995862358
0.230818567 0.826977309 0.28595339
0.64857832 0.827216035 0.538139842
0.392098397 0.613757455 0.700597946
0.347620362 0.620857693 0.180480223
0.734956108 0.342636522 0.312240901
0.583480814 0.279112744 0.831371725
0.485911005 0.358172137 0.372547759
0.114972024 0.29178989 0.101220471
0.070711346 0.298829464 0.58121134
0.976927791 0.554617547 0.908886421
0.879232808 0.633503831 0.450626929
0.958761268 0.895240805 0.903225496
0.727876993 0.569897052 0.969808502
0.503874022 0.017355211 0.378523429

EA90 3.05 4.59 4.61 76.2 109 109
1.0
3.0543031545 0 0
-1.5005018265 4.3386802135 0
-1.5156789876 0.64131085997 4.3032110685
4 12
Direct
0.46794399 0.859597144 0.82591512
0.050289519 0.325720517 0.326131099
0.400626105 0.168544355 0.177981247
0.943752245 0.666483294 0.639994718
0.790541115 0.44454932 0.795447
0.361387579 0.714827357 0.601584952
0.063894005 0.905292275 0.779942964
0.200486436 0.904119623 0.193514807
0.756546148 0.059264371 0.28114326
0.700411403 0.106564902 0.761377401
0.714426541 0.721696012 0.389366579
0.332511614 0.313334816 0.934201622
0.420993257 0.233735303 0.438777841
0.069786773 0.565684863 0.174714633
0.606364483 0.712201903 0.057697889
0.109969452 0.320976168 0.591943882

EA91 4.74 4.84 4.67 90 90 74.4
1.0
4.7411249386 0 0
1.3011923556 4.6580462182 0
0.0019087268881 0.0016056183131 4.6731875106
6 18
Direct
0.40938837 0.640812845 0.039954646
0.537755749 0.326658814 0.862852532
0.909565667 0.141067599 0.964663427
0.037659841 0.826629709 0.141597567
0.816200331 0.229613799 0.591692512
0.315879825 0.728597717 0.412837215
0.284197341 0.234437959 0.216963535
0.364214611 0.212210135 0.77592038
0.976338179 0.344043877 0.042980172
0.371264154 0.202440132 0.350808568
0.870381843 0.704200777 0.653491256
0.476589295 0.843735518 0.96195267
0.86377915 0.712477939 0.228614511
0.783998601 0.735621893 0.787823957
0.16326339 0.613640893 0.967686983
0.519601187 0.53643521 0.274338095
0.663362501 0.113347778 0.036360485
0.020415917 0.038014305 0.729928273
0.716129301 0.079580649 0.451244826
0.215709248 0.578422018 0.553197165
0.456281421 0.860717774 0.531050892
0.956036064 0.362271287 0.473272697
0.13662647 0.965651011 0.333632514
0.636804358 0.466394753 0.671303338

EA92 4.44 6.95 3.52 89.7 100 87.8
1.0
4.4375259467 0 0
0.26105993926 6.9406115139 0
-0.62540413591 0.03960955477 3.4617453839
6 18
Direct
0.316531003 0.442824819 0.10197468
0.986231234 0.550659413 0.247759753
0.795433565 0.943193422 0.897067185

0.427163499 0.056626039 0.734721015
0.769316094 0.184523156 0.718357211
0.338305737 0.675945335 0.300199689
0.114161435 0.381840726 0.294374689
0.83240214 0.595722545 0.952080517
0.798723654 0.890473415 0.214237201
0.522361493 0.351444738 0.270668502
0.954108463 0.857744911 0.717418251
0.238520822 0.398020752 0.781192908
0.329220974 0.069835538 0.01948604
0.544476552 0.893033027 0.66916814
0.801591293 0.330293125 0.885841425
0.405182928 0.606907477 0.988271764
0.322317224 0.824380394 0.138313796
0.92535971 0.10377232 0.015824773
0.858475912 0.182778006 0.427529044
0.531403651 0.657009884 0.575663153
0.245122701 0.045194923 0.451361472
0.859014469 0.505578041 0.496727882
0.094066319 0.692848454 0.452194495
0.489992429 0.232043509 0.692062586

EA93 3.53 3.42 8.5 90 90 87.2
1.0
3.526583887 0 0
0.16844178284 3.4164660429 0
-0.00081012798635 0.00081911783986 8.5013101335
6 18
Direct
0.388205741 0.921685512 0.084726377
0.605440516 0.068436019 0.58472587
0.874765629 0.857644683 0.417356358
0.119060485 0.133040454 0.917421349
0.140685589 0.148826459 0.261836072
0.852854298 0.841707362 0.761897723
0.861678821 0.315011803 0.220475089
0.131777007 0.675309938 0.720678747
0.662540737 0.741276391 0.325365335
0.331201151 0.24938186 0.825403248
0.207726529 0.975902401 0.391232737
0.786038101 0.015434698 0.891073076
0.825823078 0.179311531 0.689649068
0.168027621 0.810980788 0.189760538
0.470530717 0.766369047 0.573344041
0.523498212 0.223538922 0.073340445
0.790603864 0.210280971 0.474799919
0.202827702 0.780219416 0.974795748
0.604477547 0.639195683 0.79584008
0.389025365 0.351529677 0.295673699
0.361065204 0.316704317 0.588613208
0.632278131 0.673067664 0.088402766
0.958178849 0.381368613 0.990725497
0.035623171 0.609104805 0.49058756

EA94 5.07 4.59 4.67 90 90 107
1.0
5.0730917211 0 0
-1.3692271666 4.3761467691 0
-0.00016279453609 -0.00096977717059 4.6702581264
6 18
Direct
0.72825932 0.449929331 0.488278352
0.270251797 0.546131455 0.987976587
0.344326361 0.89649979 0.155615818
0.654091128 0.099509165 0.655441015
0.633595958 0.114392087 0.293140297
0.364893444 0.881970321 0.793241932

0.308011763 0.632669168 0.260113843
0.691505609 0.363419415 0.760317067
0.035620636 0.409201208 0.969488681
0.962772148 0.587065052 0.469266033
0.85376942 0.032026967 0.301347945
0.144909798 0.964331736 0.802046765
0.677505164 0.371074581 0.191925457
0.320132324 0.625290299 0.691754603
0.247748559 0.285294269 0.458359903
0.750763517 0.710846445 0.958316159
0.584387988 0.607113731 0.480398205
0.414039459 0.388819792 0.979320435
0.875136539 0.693599773 0.03350013
0.123191672 0.302774032 0.532989217
0.156459526 0.880067139 0.632979065
0.84209381 0.117010218 0.132752749
0.145032425 0.95459812 0.23645368
0.853192565 0.040870464 0.735825311

EA95 5 4.53 5.26 64.5 85.9 80.2
1.0
4.9992551199 0 0
0.76785799603 4.4684619324 0
0.37483329385 2.233233309 4.7451616603
6 18
Direct
0.919398684 0.462118687 0.435523319
0.637381043 0.60286063 0.205289702
0.878037505 0.27110088 0.206294892
0.376779697 0.459875333 0.826938007
0.417921315 0.878350547 0.594986311
0.13792735 0.792930147 0.826830281
0.58094263 0.601022631 0.702467476
0.587957762 0.871630421 0.019990159
0.782931392 0.751440403 0.28784768
0.093565994 0.879277738 0.010971905
0.088204028 0.627861297 0.40807525
0.347262117 0.949711956 0.36421024
0.587723912 0.016571329 0.621054042
0.762223219 0.537131934 0.009273594
0.015341665 0.233621103 0.028158562
0.761558501 0.054210055 0.335893706
0.451950858 0.483332561 0.332215401
0.258485315 0.370956865 0.699509742
0.515886161 0.245705267 0.00496435
0.262722507 0.531348642 0.023276167
0.284597923 0.023147212 0.741307392
0.08221642 0.290521445 0.329573709
0.949413786 0.801125151 0.70301961
0.849511133 0.303752212 0.666580105

EA96 4.99 4.53 5.26 64.5 85.7 80.2
1.0
4.9918837453 0 0
0.77528477188 4.4676102042 0
0.39296247914 2.2309302171 4.7512840648
6 18
Direct
0.491175984 0.877083738 0.096856248
0.451638501 0.687672659 0.866912958
0.951743567 0.879600316 0.485342316
0.991460985 0.299061627 0.255987468
0.71012571 0.21008533 0.487540661
0.21073619 0.021005628 0.864908124
0.159902079 0.438899735 0.282010332
0.022962758 0.904181903 0.989437219
0.156237887 0.023119307 0.36419172

0.164509296 0.290143575 0.679760719
0.09022901 0.66386363 0.662409224
0.355553338 0.167161357 0.950857551
0.661975183 0.293762914 0.672821642
0.660498736 0.041989643 0.070591909
0.921690348 0.369097814 0.025556206
0.337116546 0.953628266 0.669868372
0.591513534 0.64909907 0.690268638
0.33400567 0.471435285 0.995702596
0.835253599 0.793170553 0.355017296
0.83595511 0.948182831 0.682580503
0.854936339 0.4422013 0.402679339
0.655722869 0.708303885 0.989159509
0.523153629 0.216730135 0.362740138
0.421155814 0.716923072 0.327313393

EA97 4.7 3.63 6.58 75.1 80.7 76
1.0
4.7009040802 0 0
0.87536058516 3.5232329324 0
1.0642641922 1.4752427664 6.321522289
6 18
Direct
0.269322362 0.818546571 0.920535513
0.564462099 0.994156808 0.755576096
0.392459633 0.538152579 0.410461229
0.350064491 0.876622009 0.161534446
0.617556533 0.656525165 0.998702706
0.703790564 0.689655296 0.245602508
0.072618963 0.01989022 0.011053379
0.187129777 0.572605088 0.876958994
0.953125054 0.994136261 0.492178324
0.267007698 0.124750183 0.781272823
0.332734366 0.708963905 0.543439408
0.59255659 0.317648106 0.718197475
0.289336396 0.199980711 0.176914464
0.183624908 0.665956099 0.286482918
0.693121818 0.329049839 0.991647965
0.01053793 0.14794107 0.518563504
0.911347912 0.410619024 0.701930287
0.675242937 0.379139908 0.381389376
0.767807854 0.938334428 0.301601695
0.626670982 0.833999479 0.618242394
0.78467457 0.861221183 0.878164268
0.917709274 0.471333467 0.189245912
0.034053771 0.511778238 0.653430532
0.346072842 0.221609545 0.452072017

EA98 5.13 4.49 4.75 90 90 108
1.0
5.1338296458 0 0
-1.3576779364 4.2850205627 0
-0.0032826191454 0.0024323394243 4.7461536466
6 18
Direct
0.731541584 0.41434624 0.499529795
0.334712317 0.535203883 0.999275183
0.3817477 0.89403564 0.183772495
0.684626278 0.055377637 0.684058176
0.686013117 0.056939873 0.312412732
0.380192215 0.892882624 0.812051002
0.253103708 0.611134794 0.241413257
0.534059452 0.489218531 0.498834818
0.127681754 0.325024113 0.999491537
0.93834267 0.62467009 0.499367508
0.81326143 0.338054481 0.741825826
0.262749416 0.017067196 0.998094288

0.810983054 0.341169205 0.255547962
0.255140871 0.609047193 0.755115732
0.198839337 0.942186788 0.310506344
0.532264125 0.460385006 0.999061923
0.867901796 0.007096502 0.810360529
0.91292211 0.653378725 0.92546412
0.894330326 0.681071593 0.077044235
0.155385193 0.289620035 0.423499718
0.194210081 0.93674365 0.68672364
0.871559111 0.013063052 0.1865918
0.169688052 0.274510197 0.577854887
0.803730671 0.93259621 0.498980061

EA99 4.91 4.42 5.32 67.1 87.5 83.8
1.0
4.9073082944 0 0
0.47706110749 4.3963806482 0
0.23305026168 2.0545613025 4.8985603301
6 18
Direct
0.954095856 0.485587174 0.365385443
0.671926356 0.597015344 0.159062276
0.916440453 0.310452155 0.131642153
0.413009047 0.488797451 0.760541673
0.480050621 0.903470147 0.560003977
0.18125157 0.823726257 0.765183677
0.634124656 0.631718172 0.695637739
0.670957579 0.855320407 0.975445578
0.714914499 0.645382964 0.368230217
0.128673932 0.917235164 0.94361428
0.114821285 0.682623021 0.310627375
0.413967743 0.960380664 0.332439971
0.739093734 0.112873897 0.254508385
0.961121757 0.351985383 0.904343686
0.125942437 0.250715534 0.266784569
0.900131629 0.000475015 0.240518285
0.46242902 0.489409004 0.24373758
0.657139115 0.050505666 0.587168797
0.522251002 0.251058792 0.929235725
0.28265952 0.553373313 0.949268558
0.341780417 0.053865617 0.69620486
0.307545763 0.434613537 0.594498243
0.998709325 0.826570328 0.630503884
0.940216351 0.30408248 0.596640806

EA100 5.49 5.52 4.49 73.6 105 67.4
1.0
5.4875934016 0 0
2.1247189399 5.0965072528 0
-1.176839923 1.8677259835 3.9131985486
6 18
Direct
0.386397103 0.658386159 0.980424969
0.619791726 0.339070464 0.01646101
0.04804399 0.615456268 0.400238688
0.957438928 0.381872206 0.597100883
0.665486653 0.951351321 0.638280041
0.34082595 0.04618379 0.357737346
0.472878258 0.795484484 0.082407112
0.13587947 0.757979422 0.840772982
0.870336177 0.239718822 0.156065399
0.53375404 0.201846856 0.914014414
0.894557053 0.866326305 0.276883319
0.728477152 0.468444083 0.602602882
0.11173615 0.131236371 0.719751432
0.277334341 0.527811295 0.39578386
0.768521394 0.100496317 0.599326763

0.236568441 0.898158148 0.394960917
0.800801171 0.725295096 0.875559991
0.206349626 0.272779797 0.12146835
0.412128554 0.090571198 0.626237456
0.594214121 0.904367059 0.371542933
0.504570239 0.595083648 0.7803392
0.50054935 0.402770262 0.216059494
0.089646021 0.504107378 0.715234698
0.914569593 0.49421587 0.281805104

EA101 4.72 4.8 4.82 74.9 90.1 90.1
1.0
4.7216506636 0 0
-0.0078015453976 4.800402586 0
-0.0063858761339 1.251844465 4.6501411943
6 18
Direct
0.348997595 0.678409644 0.764326393
0.34444243 0.44322608 0.534682682
0.582329585 0.053344009 0.153393513
0.082240378 0.708558498 0.50385156
0.845945827 0.31762067 0.126976743
0.847193883 0.088859329 0.891116948
0.040067881 0.236717552 0.977527528
0.017803477 0.907648955 0.90221945
0.070594169 0.907381979 0.300522507
0.22049857 0.898150332 0.589743054
0.714984232 0.14141893 0.344035105
0.521892323 0.858641889 0.749453114
0.213729408 0.615652819 0.316005889
0.512788511 0.452178664 0.352630316
0.250064491 0.216739021 0.644062383
0.540301528 0.528115112 0.6799107
0.36845967 0.151151254 0.056399787
0.256101579 0.573421533 0.9922127
0.754342844 0.197386727 0.664293133
0.01641298 0.304040287 0.308412227
0.573496837 0.847295453 0.349895397
0.751813666 0.545508992 0.022666711
0.869625438 0.611894892 0.605617073
0.718957079 0.866877162 0.062249946

EA102 4.81 5.66 4.9 66.4 61.5 70.3
1.0
4.8061591189 0 0
1.9128356123 5.3305119229 0
2.3398363867 1.2429071852 4.1219291947
6 18
Direct
0.001611161 0.062759215 0.512228167
0.19205755 0.067017487 0.066207249
0.426865675 0.738062534 0.059977625
0.448925911 0.485509705 0.404680961
0.714877926 0.560928642 0.788165142
0.785943877 0.602205915 0.080871597
0.052633708 0.268453915 0.457703804
0.939806222 0.015046403 0.314568696
0.308327864 0.25041802 0.977831906
0.087307461 0.085987536 0.883065478
0.174024833 0.881474669 0.609912604
0.655966291 0.046552073 0.195946556
0.473628002 0.945860176 0.058029849
0.540275943 0.988736761 0.361312961
0.353450776 0.565284848 0.624375454
0.407991941 0.720177722 0.826163051
0.214128188 0.615571525 0.282464924
0.395118197 0.283566947 0.455953656

0.76822735 0.406845428 0.325481181
0.724859277 0.058695576 0.672676707
0.969639426 0.481669221 0.875717094
0.645313406 0.35593123 0.872076868
0.837165768 0.665605844 0.511101457
0.928650164 0.738594631 0.068160015

EA103 4.81 4.83 4.59 86.4 93.5 102
1.0
4.8077333976 0 0
-0.98148423688 4.7308505014 0
-0.283092658 0.23268714537 4.5761508293
6 18
Direct
0.142161264 0.44610725 0.004956525
0.085863175 0.155135115 0.78014186
0.863641782 0.408288437 0.727462088
0.231814159 0.010963594 0.514828818
0.519926755 0.065140284 0.283497972
0.48566787 0.789133951 0.563040587
0.707515788 0.259459425 0.246306936
0.669826142 0.596220276 0.14721499
0.643647305 0.308664209 0.820829893
0.019787674 0.622191508 0.84893094
0.862476629 0.558509215 0.516152115
0.426491942 0.963597097 0.067272372
0.314067883 0.315160977 0.881425945
0.043126823 0.360659656 0.225099329
0.69785516 0.940718901 0.434613786
0.381562878 0.569058338 0.474185787
0.973374543 0.266989004 0.554580704
0.340148324 0.631170807 0.036022285
0.352536263 0.904960467 0.738926978
0.049008667 0.882954842 0.365122275
0.641479656 0.789988535 0.77097077
0.389616088 0.237140648 0.407819627
0.79666587 0.714523685 0.145599258
0.961436355 0.973917147 0.934054691

EA104 4.72 4.83 4.81 73.9 90 90.1
1.0
4.7196952948 0 0
-0.00775662299 4.8258697679 0
-0.002214324599 1.3325726135 4.6254331785
6 18
Direct
0.933646105 0.109263014 0.546916103
0.912324864 0.850351784 0.35222995
0.147458121 0.458042785 0.953496532
0.65867185 0.121696026 0.297410982
0.425552895 0.724395362 0.932451295
0.406030557 0.509666175 0.673888215
0.63929512 0.287509306 0.067096189
0.791812942 0.319777778 0.369129829
0.313412065 0.523837639 0.136626415
0.12550065 0.272401896 0.506634405
0.788430272 0.009217604 0.12171986
0.075328093 0.8367705 0.171469315
0.820911939 0.62695512 0.483668718
0.119894131 0.952163364 0.461675546
0.134501789 0.531974498 0.679059507
0.382392681 0.632260497 0.428750161
0.164965175 0.210126499 0.058459156
0.446902054 0.036672257 0.410643893
0.314794161 0.950266713 0.857273279
0.520110729 0.286885087 0.725544392
0.585249963 0.686015196 0.733969715

0.929476149 0.574020648 0.950945394
0.616027401 0.702253861 0.093944243
0.850611687 0.023847041 0.784713273

EA105 4.85 4.85 4.86 100 99.9 100
1.0
4.8543612745 0 0
-0.85496492049 4.7714955446 0
-0.83855873738 -1.0211268456 4.6733780408
6 18
Direct
0.877199142 0.1399217 0.072855045
0.076261005 0.877563929 0.13733743
0.141117181 0.076443069 0.874809285
0.575744105 0.642160617 0.376240766
0.376566326 0.574760456 0.63822293
0.641197576 0.378882105 0.573402002
0.645228853 0.398184984 0.313668061
0.31551657 0.645425313 0.395439772
0.39698066 0.314568491 0.641053755
0.815372746 0.894248815 0.139300084
0.146213905 0.816157583 0.893854003
0.897258121 0.145362747 0.813511363
0.839275067 0.490128193 0.754233271
0.755898384 0.841923367 0.486222106
0.485436133 0.754602447 0.838354112
0.256021419 0.988820662 0.33642103
0.339730268 0.257787483 0.985134617
0.98647911 0.338935706 0.254154767
0.619301563 0.130511164 0.49025447
0.494043566 0.618111025 0.128077521
0.128425893 0.490467034 0.614089761
0.995238598 0.628439666 0.113363043
0.117881455 0.993672111 0.626587447
0.628936733 0.115653279 0.990858031

EA106 3.62 3.61 9.09 88.6 86.2 60.2
1.0
3.6228520031 0 0
1.7947593896 3.1333060877 0
0.6072432831 -0.094435486107 9.0654939524
6 18
Direct
0.395341271 0.978090999 0.13897434
0.734183153 0.000565226 0.978186485
0.050015472 0.673643173 0.810849484
0.354019446 0.725968238 0.643095514
0.307686526 0.064826307 0.47908591
0.013991392 0.330488207 0.304055803
0.613721261 0.366668219 0.649271999
0.656037586 0.005999966 0.464592887
0.102813642 0.597714753 0.312318996
0.410031605 0.647977488 0.141784975
0.336076452 0.109239227 0.002806177
0.034159192 0.35876732 0.808439756
0.00019568 0.247917918 0.16481748
0.774298834 0.298259227 0.989166044
0.665778784 0.992818844 0.840681669
0.967238819 0.820003741 0.677489138
0.275393451 0.724915855 0.507035586
0.24763817 0.951886231 0.352698525
0.660654448 0.413997018 0.331716398
0.640532764 0.02238103 0.203869032
0.98282695 0.674697032 0.027386008
0.345904307 0.670858333 0.858438742
0.439268922 0.980947239 0.67938656
0.024188073 0.386540998 0.527734897

EA107 4.43 4.45 6.64 89.1 71.1 60.8
1.0
2.22656482 3.8287508605 0
-2.261976374 3.8287508605 0
2.0184016166 1.3116949473 6.192811613
6 18
Direct
0.17637149 0.244070671 0.266467533
0.51511185 0.739021389 0.753392471
0.293836231 0.184127283 0.745276657
0.97277156 0.689526543 0.240493699
0.043468504 0.95801283 0.761854239
0.72996436 0.470926601 0.241122666
0.275579622 0.315558564 0.902818133
0.802202616 0.670235991 0.119019531
0.417832356 0.089555951 0.102004655
0.287135065 0.643134665 0.762640705
0.915812931 0.183722552 0.303949536
0.576295393 0.707804529 0.913464686
0.968635531 0.275823261 0.756718131
0.179834762 0.729159276 0.096953955
0.155657038 0.501264904 0.360307887
0.602666524 0.919066476 0.619614202
0.350744666 0.313726261 0.590971547
0.769184148 0.931725103 0.379959577
0.22617958 0.10920055 0.414594951
0.736115906 0.512730621 0.603865721
0.463174516 0.613341098 0.398578602
0.015100443 0.899342394 0.598118968
0.802816952 0.003680856 0.921791146
0.705753319 0.345936252 0.098102633

EA108 4.85 4.49 4.99 78.1 87.3 81
1.0
4.8514742482 0 0
0.70202353038 4.4311970708 0
0.23752462274 1.00346319 4.8800637979
6 18
Direct
0.713103745 0.209145739 0.883031505
0.334623456 0.467203186 0.404362572
0.408971171 0.387259812 0.74430403
0.264358075 0.790346611 0.175485786
0.998159403 0.557910585 0.26469497
0.353898456 0.807464008 0.502534629
0.549845712 0.123169645 0.726089218
0.88516658 0.027634492 0.479818145
0.117401614 0.353289342 0.482328952
0.806088618 0.156143144 0.382866987
0.936926684 0.214441518 0.784878359
0.227737867 0.345909312 0.903106225
0.682387385 0.065117413 0.10357182
0.501303047 0.31285671 0.295436141
0.799631511 0.773931199 0.82857265
0.4130212 0.801079635 0.985663515
0.251265121 0.024829781 0.273992041
0.185090806 0.895393292 0.657740722
0.013414678 0.846814159 0.112704707
0.952098732 0.410266153 0.108067815
0.594389125 0.494548821 0.856460025
0.912234379 0.699380535 0.746375888
0.573775163 0.875240772 0.517590618
0.805004875 0.639100988 0.396805526

EA109 4.66 4.62 5.1 86.9 100 92.5
1.0

4.6635575112 0 0
-0.20381885787 4.6176771493 0
-0.9104932598 0.23507874285 5.010743366
6 18
Direct
0.394407508 0.097150184 0.162769035
0.633190652 0.872303863 0.09328422
0.065041484 0.560615976 0.447726992
0.029523016 0.216181218 0.603932172
0.435063506 0.599700862 0.594965785
0.155088079 0.530358324 0.773835047
0.226653224 0.070539542 0.589760564
0.978741373 0.329867254 0.340763737
0.633317207 0.009575015 0.307404895
0.845713077 0.923442591 0.0058325
0.793600054 0.445695058 0.013010926
0.431271739 0.344712015 0.178737074
0.277562773 0.604657266 0.334514989
0.552631971 0.635475943 0.134097777
0.177917228 0.988806013 0.208235982
0.542770859 0.834217473 0.619944963
0.803956284 0.099024549 0.622185331
0.424236913 0.508746923 0.842716342
0.897911098 0.743971115 0.398232253
0.583675924 0.412594528 0.560448301
0.106064829 0.280906565 0.863166037
0.763840182 0.294195962 0.973807517
0.434237902 0.020075532 0.933763549
0.063050864 0.697935904 0.898043977

EA110 4.6 4.91 4.81 75.2 90.9 91.1
1.0
4.5997879062 0 0
-0.095045751142 4.9104078146 0
-0.078253402598 1.2295758638 4.6455343818
6 18
Direct
0.357018931 0.59675902 0.79059969
0.344202663 0.361187642 0.571241703
0.592040851 0.953949574 0.183565054
0.07971458 0.619973259 0.531711979
0.855313417 0.215830931 0.157581741
0.84014104 0.016618255 0.913760265
0.996482037 0.228886028 0.922656773
0.99216641 0.820649201 0.950908261
0.764327148 0.44711275 0.103925395
0.223363984 0.807421637 0.619355935
0.627403756 0.042805531 0.392141034
0.580448626 0.709791538 0.256936057
0.053336298 0.793949734 0.314518938
0.555941467 0.472077321 0.685871555
0.258595826 0.133800094 0.687136754
0.331404129 0.049698652 0.167090833
0.512869978 0.367062679 0.388706231
0.546579483 0.763259702 0.785445643
0.749248991 0.104160079 0.679275262
0.193683811 0.503721156 0.353508327
0.035278065 0.163614015 0.338720614
0.401097797 0.070629124 0.991523093
0.265103647 0.489710683 0.019599751
0.863870335 0.535505392 0.64172519

EA111 4.84 4.66 4.78 89.8 102 89.9
1.0
4.8373686696 0 0
0.0075932309517 4.6570055772 0
-1.0343558746 0.016685435294 4.6713410617

6 18
Direct
0.696067505 0.046170994 0.571364096
0.299144384 0.798124905 0.930168036
0.069183323 0.799763812 0.174306866
0.334775308 0.528285034 0.205745067
0.742558722 0.311378494 0.849169652
0.974140661 0.313716801 0.61141791
0.035019114 0.856293098 0.905219131
0.082005829 0.532532786 0.244790888
0.75205166 0.824639678 0.680314655
0.526922408 0.212661031 0.658301372
0.522746299 0.584757079 0.394833347
0.472141859 0.96945091 0.015397486
0.171044851 0.967763837 0.345611225
0.518932269 0.034474893 0.361600515
0.820752697 0.774714636 0.140921143
0.874422465 0.118578391 0.426660245
0.29523885 0.279105676 0.166211064
0.015634119 0.298765908 0.884577777
0.652206778 0.544276262 0.81594227
0.254264061 0.766572119 0.679228188
0.903023771 0.531740467 0.498378342
0.760133857 0.211611694 0.077415514
0.371933272 0.532134544 0.949865872
0.213412694 0.248706961 0.638812851

EA112 4.81 4.72 4.81 90 105 90
1.0
4.8127470567 0 0
-0.0010724276927 4.7187612042 0
-1.2461225139 0.00020545347132 4.6423510118
6 18
Direct
0.685204343 0.077901953 0.950279011
0.07403926 0.312924898 0.572537608
0.81162428 0.578022146 0.541413065
0.422757366 0.812751688 0.919211822
0.04389484 0.576930049 0.308900437
0.452867278 0.076855912 0.182676142
0.600595338 0.271007093 0.098742291
0.262616088 0.447255754 0.482335717
0.896419931 0.771024962 0.392907291
0.234085276 0.947374732 0.008728845
0.521030706 0.599562527 0.017905579
0.825208467 0.749536566 0.722288715
0.975395879 0.099756786 0.474099723
0.67176695 0.249168805 0.769197953
0.222605481 0.802339856 0.717290512
0.584020843 0.485501874 0.434899985
0.274393506 0.302544084 0.774240263
0.9127191 0.985345172 0.056888031
0.984123206 0.44819905 0.760820697
0.512765119 0.947774081 0.730885479
0.224735062 0.74776024 0.32041646
0.559763316 0.98249481 0.409505811
0.27195541 0.247491298 0.171277446
0.936977264 0.482999129 0.081915715

EA113 4.54 4.72 5.03 100 94.7 92.6
1.0
4.5418635107 0 0
-0.21595942186 4.7158934774 0
-0.41356824537 -0.93305137037 4.9232598957
6 18
Direct
0.834736393 0.003127326 0.972943582

0.197887335 0.984535799 0.07371057
0.084335886 0.222017817 0.867525229
0.650255161 0.643297645 0.389035661
0.291200557 0.611561777 0.489028898
0.570244804 0.41754336 0.620891762
0.705796924 0.053783222 0.197223651
0.215547454 0.085371426 0.306197957
0.347715786 0.548579569 0.722828912
0.648961777 0.931360542 0.799147176
0.343001818 0.178991385 0.956646696
0.073684069 0.465782304 0.96609401
0.806725716 0.556055965 0.582428071
0.809129103 0.855237714 0.438097163
0.65262749 0.511118245 0.166874946
0.131538678 0.466625114 0.32136905
0.437744859 0.820589424 0.429366814
0.654325011 0.460643164 0.853122373
0.546728097 0.174930426 0.517996544
0.763744108 0.217579361 0.157559287
0.926728272 0.792501247 0.053906967
0.339339774 0.785097883 0.015993954
0.065332905 0.164341935 0.626912907
0.181526031 0.805070592 0.620409324

EA114 5.32 3.28 4.96 90 90 101
1.0
5.3228559353 0 0
-0.64826867167 3.2165489406 0
0.00091052303061 -0.00029418405437 4.9589415226
6 18
Direct
0.266351478 0.640308836 0.016975838
0.237388401 0.359108476 0.517054173
0.803393784 0.664363383 0.738602137
0.700220711 0.334491501 0.238575644
0.040729025 0.309576013 0.235329272
0.463161509 0.689865601 0.735313552
0.609810681 0.149613117 0.05168872
0.894168935 0.849905363 0.552108772
0.355379872 0.205198369 0.363336842
0.148213333 0.794319963 0.86361284
0.041075225 0.501566758 0.472735066
0.462718735 0.497929536 0.972464726
0.902639058 0.540345126 0.137254857
0.601279236 0.459845952 0.636270544
0.808487953 0.867510617 0.933051339
0.694742023 0.130852824 0.432881513
0.342582911 0.845575339 0.198550939
0.161276291 0.15346153 0.698567027
0.341805521 0.744473944 0.522068087
0.161857191 0.255255474 0.021634783
0.64104961 0.655113497 0.271125328
0.862508773 0.343741401 0.770994939
0.980184585 0.953554202 0.23927931
0.523350316 0.045940077 0.73942125

EA115 4.57 3.18 6.52 89.9 101 110
1.0
4.5691118333 0 0
-1.0959604099 2.9899876523 0
-1.2064709388 -0.42716936028 6.3884800365
6 18
Direct
0.557986558 0.176422613 0.262102795
0.219115906 0.496780195 0.594629118
0.790551064 0.924358373 0.378395208

0.45169115 0.244940485 0.711039121
0.883600678 0.977622797 0.92547063
0.126666372 0.444286275 0.047609297
0.749115749 0.775721957 0.536673091
0.415932717 0.11082559 0.872662423
0.355840031 0.200473283 0.339630998
0.016546781 0.517718055 0.672025128
0.653489885 0.220073214 0.633374914
0.594432138 0.311263326 0.100653817
0.476046145 0.652723165 0.72553107
0.533593628 0.768670401 0.247611826
0.99325249 0.904185409 0.300933028
0.189064012 0.08506303 0.581932345
0.820016355 0.335698629 0.391767324
0.260153274 0.64450886 0.436155277
0.919182786 0.875303841 0.76488152
0.141842309 0.055018786 0.053838497
0.682410701 0.755217458 0.003706578
0.868856163 0.367069788 0.918763697
0.090046094 0.545560523 0.208066019
0.328472672 0.667246405 0.969957086

EA116 4.56 3.35 6.21 78.3 90.9 71.5
1.0
4.564149347 0 0
1.0651641359 3.1751657373 0
-0.093811232371 1.357897258 6.0549642371
6 18
Direct
0.606253226 0.31327884 0.126878067
0.445515234 0.490641767 0.35064479
0.804071239 0.140601121 0.368777811
0.956653391 0.378021656 0.156263629
0.098543226 0.887520991 0.801828777
0.229596499 0.427151261 0.722059553
0.378353839 0.628644163 0.133925641
0.304494656 0.274590759 0.422602175
0.019862909 0.277636967 0.374748062
0.981864762 0.715107912 0.140025021
0.379213575 0.829624389 0.386904494
0.179865557 0.822853311 0.616280462
0.653384286 0.814031974 0.663140959
0.546781793 0.053087632 0.057210144
0.691293389 0.364614921 0.500555315
0.156709941 0.111601157 0.108344595
0.866023779 0.778458425 0.430876088
0.724375624 0.519774933 0.98898098
0.30289072 0.93808853 0.896992514
0.838344524 0.048121532 0.824179453
0.034294802 0.370725018 0.620861703
0.14185904 0.487034695 0.904798317
0.493365335 0.264606012 0.724677606
0.651489448 0.602186839 0.724326185

EA117 4.65 2.97 6.59 90.2 98.9 108
1.0
4.6508105304 0 0
-0.9426702658 2.8199049127 0
-1.0229895692 -0.36253875487 6.4962024284
6 18
Direct
0.007822079 0.773176503 0.939451794
0.338193178 0.423517789 0.607830161
0.541054454 0.028293122 0.705293527
0.204925278 0.37713707 0.041905109
0.666556349 0.151270958 0.271343813
0.87153634 0.744901027 0.379049428

```
0.104506039 0.190573193 0.647553751
0.216964137 0.118951674 0.896915578
0.093091649 0.796667295 0.310913368
0.580129238 0.196255135 0.870896043
0.445135131 0.614515713 0.013904262
0.2979373 0.524162304 0.438584635
0.003666007 0.529510708 0.092484
0.519718584 0.245098808 0.545567434
0.346524254 0.679711646 0.760318233
0.432128732 0.946906222 0.318793686
0.224984621 0.271613862 0.211116804
0.887959101 0.647616748 0.550050332
0.774919158 0.019431396 0.671571508
0.773688607 0.784123628 0.973183947
0.904611922 0.180652587 0.401559171
0.971519605 0.616885004 0.772602431
0.613753103 0.546929079 0.269920049
0.678009951 0.123320056 0.098678937

EA118 4.66 3.01 6.54 90.8 99.7 71.4
1.0
4.6563277633 0 0
0.96253064213 2.8526295678 0
-1.0994971179 0.27823591291 6.4441234983
6 18
Direct
0.062512824 0.062941738 0.932905713
0.395049168 0.370897308 0.597500659
0.592729997 0.782207071 0.699770625
0.260463747 0.474364841 0.034914495
0.706161897 0.653764556 0.252050153
0.949174338 0.191087372 0.380461246
0.15639759 0.36637609 0.62890947
0.262802212 0.214023485 0.884642586
0.024528295 0.936048298 0.765284172
0.160184206 0.97864274 0.311725225
0.630772007 0.90768799 0.867622459
0.829765459 0.309271153 0.965453846
0.499205301 0.478253063 0.003461513
0.371625535 0.505057969 0.428671379
0.069992725 0.83037687 0.091618994
0.586182465 0.015059218 0.541364949
0.825415907 0.535647272 0.66712372
0.391373754 0.63258012 0.747356699
0.739646798 0.793610311 0.096807212
0.709560289 0.235770335 0.24538808
0.494994021 0.86677163 0.32050045
0.945843694 0.608973025 0.38710787
0.916293718 0.050504545 0.535750761
0.28377436 0.340159031 0.203719719

EA119 4.61 3.04 6.58 89.8 100 109
1.0
4.6071596416 0 0
-0.98766429918 2.8790054628 0
-1.1375840545 -0.37139241366 6.4686303285
6 18
Direct
0.052044424 0.749721928 0.940606561
0.345022106 0.50784457 0.593167961
0.608825511 0.954182273 0.724428621
0.258042417 0.347397991 0.04097766
0.695864294 0.11460877 0.276557761
0.901899086 0.712443874 0.376914519
0.159155477 0.25673032 0.673513591
0.332591756 0.020418644 0.979352579
0.103702357 0.795442621 0.286645421
```

0.349855851 0.921150035 0.608310879
0.519371592 0.539223475 0.034607173
0.342584672 0.396870564 0.425691615
0.85022995 0.666411339 0.030879956
0.603840403 0.540761632 0.70940423
0.121424691 0.430381994 0.867062083
0.434593032 0.922854654 0.283150677
0.185053685 0.341156896 0.199682542
0.970438147 0.66574104 0.553554969
0.794699503 0.205317499 0.644110578
0.611554376 0.065466444 0.891916924
0.832902304 0.032206523 0.450426411
0.983438302 0.796486955 0.763965265
0.621446213 0.441593529 0.338298417
0.768831685 0.120800131 0.117845024

EA120 4.37 3.19 6.69 95.1 98.5 110
1.0
4.3693582243 0 0
-1.0877101092 2.9936543084 0
-0.98378966107 -0.99122323549 6.5388128111
6 18
Direct
0.308589178 0.228353673 0.260465315
0.187571839 0.307637026 0.672781514
0.541484419 0.228438741 0.083337684
0.534612254 0.267617075 0.820095425
0.861147521 0.902753911 0.506294404
0.150449874 0.873062878 0.042148123
0.661422086 0.06841198 0.40829729
0.891748951 0.124418492 0.68566038
0.197183693 0.498220478 0.304950816
0.602277634 0.684553111 0.53660523
0.571343566 0.550145553 0.98008562
0.862467125 0.143808365 0.367514942
0.275673282 0.539400704 0.554223319
0.152087615 0.518265566 0.813932696
0.723195362 0.524516119 0.744297023
0.624925328 0.963697003 0.824410147
0.982578449 0.982405185 0.923106935
0.975705397 0.66980836 0.428779046
0.396328082 0.093395851 0.40841404
0.760125057 0.111461899 0.130050012
0.284914687 0.984303393 0.698770887
0.590357366 0.543576963 0.221370967
0.111925272 0.494027663 0.02841137
0.025288831 0.873722741 0.194383598

EA121 4.59 3.09 6.54 90.3 100 70.7
1.0
4.5895167125 0 0
1.0202258528 2.9208305377 0
-1.1518552544 0.37010823619 6.4228636212
6 18
Direct
0.301107859 0.379085048 0.275173414
0.386690632 0.17140716 0.817353688
0.736581898 0.578254794 0.498962085
0.625810687 0.696920849 0.938826197
0.93767547 0.980835377 0.59191408
0.052537812 0.836295328 0.145716318
0.408816752 0.316150957 0.660145788
0.383636825 0.763370307 0.801727927
0.70070048 0.463268075 0.328608415
0.191318874 0.372014614 0.903767918
0.49977464 0.80183824 0.538883844
0.574727081 0.598884471 0.095354121

```
0.489698705 0.120996012 0.197799049
0.906288413 0.768914784 0.429680549
0.847832675 0.068182666 0.218606949
0.178322063 0.964767409 0.553427775
0.967328151 0.825235602 0.758741938
0.262856943 0.310745197 0.438783804
0.297039748 0.794060145 0.275678736
0.088354913 0.934823053 0.98459438
0.650663406 0.088684449 0.941737039
0.840198023 0.458781142 0.876286676
0.031143008 0.445585901 0.154699249
0.762496072 0.334279261 0.658399959

EA122 3.89 3.89 6.67 90 90 120
1.0
1.9447191722 -3.3680520863 0
1.9447291158 3.3680520863 0
8.2380889242e-05 1.6070308773e-05 6.6668350861
6 18
Direct
0.735642399 0.768725868 0.570754589
0.23129474 0.965891956 0.570670341
0.034111181 0.264293432 0.570664262
0.931664586 0.563887 0.429880746
0.436128624 0.366787969 0.42984816
0.633261237 0.06827302 0.429806417
0.31749647 0.352920552 0.267108387
0.647130873 0.963462016 0.267073634
0.036640624 0.682875304 0.267183386
0.020339389 0.369309951 0.733379708
0.630882041 0.650210568 0.733500783
0.350109794 0.979671815 0.733369745
0.031493045 0.726565654 0.857247253
0.273249048 0.302782253 0.857270496
0.697265042 0.969537444 0.857513849
0.710191187 0.764795094 0.063188748
0.234657829 0.944879131 0.063407786
0.055259313 0.289837071 0.06325069
0.971305602 0.364766294 0.143394493
0.635936603 0.605391662 0.14301305
0.394483921 0.028522971 0.143054033
0.431725966 0.389045255 0.937515737
0.611377867 0.043079766 0.937290524
0.957609104 0.567854989 0.937423307

EA123 4.32 3.15 6.64 85.4 97.7 75.7
1.0
4.3171461253 0 0
0.77874913246 3.0514983766 0
-0.88816875085 0.77765606227 6.5392146677
6 18
Direct
0.914660621 0.424419157 0.329202901
0.201723229 0.233726839 0.165499124
0.486529639 0.323031095 0.007755095
0.169863755 0.516531999 0.543789991
0.317955218 0.207654648 0.766738278
0.560049776 0.245244777 0.59991343
0.680929984 0.705342486 0.321432011
0.831211205 0.114005247 0.291031757
0.327448012 0.549198816 0.848659316
0.347728732 0.543906688 0.13011289
0.069691945 0.194612032 0.491771351
0.687673732 0.500905123 0.0096533
0.354058395 0.985740207 0.301378809
0.907737243 0.621797076 0.861359416
0.003868407 0.812864018 0.600694024
```

0.610534352 0.951574399 0.052102481
0.06840277 0.085211766 0.038830211
0.630515535 0.990248589 0.488420352
0.119012013 0.038423029 0.794751083
0.74970747 0.413244393 0.674285188
0.560384291 0.921622945 0.751926586
0.372223215 0.613913756 0.452807075
0.017735961 0.614464519 0.191444239
0.913765921 0.831143678 0.906324779

EA124 4.12 4.53 4.79 75.7 96.4 91.6
1.0
4.1230090733 0 0
-0.12663161624 4.5315722775 0
-0.53258031498 1.1694019804 4.614615016
6 18
Direct
0.644870138 0.617138839 0.050255334
0.705611633 0.261495871 0.570948411
0.303639127 0.741641332 0.428094638
0.364004789 0.385785162 0.948809467
0.390389682 0.419146912 0.295880494
0.618750871 0.58385894 0.703217661
0.152729943 0.505448032 0.397854538
0.085206444 0.813513642 0.529676974
0.68662817 0.074479156 0.778463436
0.903655155 0.562204984 0.160972905
0.846138445 0.183675264 0.10041844
0.422758787 0.174104723 0.490712934
0.171780847 0.26582983 0.22307162
0.425659841 0.132951971 0.979707489
0.856341918 0.497811892 0.600886048
0.923939608 0.19000072 0.469042671
0.323000009 0.928752971 0.220694751
0.105169209 0.440662586 0.838153931
0.102711059 0.978355903 0.839334081
0.58646503 0.828988439 0.508541184
0.837651086 0.737013002 0.775773566
0.583260764 0.870089309 0.018691772
0.906304798 0.024247853 0.159680048
0.162829114 0.818985334 0.899191033

EA125 4.44 3.28 6.21 82.9 101 105
1.0
4.4422408034 0 0
-0.84960182631 3.1642812973 0
-1.1470574866 0.48834906263 6.0853395659
6 18
Direct
0.622758297 0.069694939 0.306466724
0.906117487 0.412276304 0.486589773
0.264815666 0.352200805 0.630081076
0.288032052 0.231474462 0.150022148
0.931605779 0.120380713 0.742520055
0.284668438 0.067420831 0.885489512
0.535839341 0.417540077 0.263507016
0.49516936 0.813921099 0.424655351
0.913471329 0.221931832 0.324922122
0.163790871 0.488813667 0.174073001
0.692566971 0.474715062 0.547627526
0.314448506 0.183542397 0.48984616
0.039402043 0.741898996 0.414874642
0.363600289 0.412508776 0.975586922
0.121766104 0.900615997 0.177362222
0.796856318 0.48728994 0.037069541
0.683775375 0.941023626 0.157373219
0.390733861 0.700698903 0.655862952

0.771202714 0.705879461 0.023319464
0.035154381 0.783710515 0.83894212
0.724361264 0.836019524 0.690837455
0.4451289 0.832887386 0.929615817
0.480843719 0.184422994 0.755851817
0.874216225 0.286502926 0.879961845

EA126 4.33 3.2 6.41 91.5 100 83.5
1.0
4.3298254863 0 0
0.36410158569 3.1792775765 0
-1.141314825 -0.041154017954 6.3057001086
6 18
Direct
0.06092326 0.722199217 0.894476488
0.941665937 0.160800092 0.283386321
0.211248754 0.475844772 0.686576447
0.457193966 0.629331668 0.906530292
0.348522526 0.057093383 0.286384614
0.661571081 0.1802319 0.485032843
0.505275855 0.562059399 0.723338533
0.943869162 0.686378205 0.705422569
0.285783581 0.95473693 0.950195426
0.47428511 0.751784474 0.227964778
0.236408528 0.273351968 0.142032278
0.194851593 0.611627005 0.518976254
0.189730612 0.995316347 0.416667692
0.931802262 0.066548987 0.880119401
0.835340595 0.970725403 0.137329908
0.606846666 0.991441462 0.623347839
0.878998855 0.521170695 0.274055249
0.71734856 0.510037562 0.544678538
0.566571317 0.38140495 0.032777003
0.595725821 0.922219589 0.907084989
0.97616277 0.507814386 0.008659232
0.864136777 0.928832415 0.42670785
0.464471919 0.389439792 0.347710496
0.190156331 0.122384908 0.674490408

EA127 4.36 3.27 6.53 93.9 97.2 68
1.0
3.1366604878 3.0281842499 0
-1.2250653382 3.0281842499 0
-0.47769441981 -0.6780430416 6.4803405662
6 18
Direct
0.260701687 0.494760314 0.834846808
0.423621416 0.077349022 0.260337139
0.79151611 0.163138665 0.214910484
0.068462109 0.322188048 0.404337526
0.57909618 0.377953596 0.668886717
0.87198315 0.479004602 0.853555753
0.376693269 0.015745749 0.086517147
0.24825579 0.462567802 0.303597858
0.983942665 0.843201051 0.150110308
0.622006299 0.39713166 0.087071819
0.110725174 0.07187148 0.533363537
0.025921965 0.260693522 0.989553774
0.991969309 0.675155989 0.482083399
0.314679106 0.719008167 0.704941575
0.002214017 0.560304738 0.7097049
0.689369499 0.796269702 0.925004909
0.434904413 0.779207868 0.351722323
0.826372673 0.13681685 0.78984937
0.677710003 0.093527914 0.370930187
0.608731379 0.585243695 0.540241767
0.255937108 0.718559104 0.979609281

0.425029638 0.128429685 0.864471647
0.822517416 0.513745837 0.272167895
0.587329205 0.040391311 0.595643905

EA128 4.62 3.04 6.53 90.1 99.7 71.1
1.0
4.6249542395 0 0
0.984168983 2.8742287459 0
-1.095811459 0.35979479286 6.4316600259
6 18
Direct
0.505392939 0.696558902 0.207175418
0.158707272 0.259589401 0.532488407
0.717552687 0.095530451 0.313543826
0.3975959 0.787544913 0.658180139
0.84231468 0.966144473 0.875911459
0.039684088 0.37199159 0.975106594
0.685461103 0.036834449 0.481564418
0.371029341 0.638296416 0.81459499
0.286840635 0.780742284 0.279858162
0.938890386 0.49254743 0.590135192
0.822113965 0.116052704 0.709100118
0.486133753 0.588331067 0.035609745
0.392366186 0.207876719 0.670467436
0.522010252 0.086737848 0.154809625
0.213443504 0.351720048 0.37547071
0.139740782 0.858324716 0.533056718
0.759054726 0.489054856 0.323537737
0.596879176 0.587245585 0.575554228
0.023407246 0.610173705 0.814425276
0.8659369 0.179875622 0.037815354
0.601411926 0.968189325 0.905841943
0.274482318 0.145313578 0.934873495
0.962109387 0.919783392 0.271864018
0.07524986 0.477160643 0.145258094

EA129 4.85 4.24 4.56 90 90 111
1.0
4.8487780971 0 0
-1.5126458377 3.9560272737 0
-0.0012840869213 0.0011965823971 4.5561168336
6 18
Direct
0.714768548 0.716968582 0.9975387
0.078806439 0.669298188 0.027755073
0.295546211 0.000886915 0.775555889
0.284241156 0.286768031 0.497320483
0.920069645 0.334586895 0.527742211
0.702834325 0.003075466 0.275398386
0.212356784 0.80775052 0.227503863
0.326267783 0.210571167 0.947422437
0.190802277 0.498936588 0.594998031
0.78724045 0.196137572 0.727873756
0.670675686 0.793472394 0.447778625
0.808383696 0.50513411 0.095689057
0.160633099 0.976244647 0.526336837
0.28561809 0.262032689 0.245514794
0.947142237 0.239026812 0.276126658
0.838914634 0.027218107 0.026868694
0.713044809 0.741434356 0.745708775
0.05162035 0.764462332 0.775993098
0.473927806 0.889687758 0.725783922
0.520353726 0.431424335 0.581257979
0.883614498 0.585332275 0.478577442
0.525208121 0.115147149 0.224256833
0.478773566 0.572376106 0.081651673
0.114453758 0.418235129 0.978553148

EA130 4.2 3.2 6.68 94.2 103 78.8
1.0
4.195217125 0 0
0.62229186619 3.1351350545 0
-1.455591333 -0.21117628375 6.5122305502
6 18
Direct
0.455081987 0.828467607 0.2470418
0.093369907 0.068794035 0.341767915
0.754859001 0.019726995 0.131397329
0.451165204 0.776544953 0.737135326
0.117485529 0.125781948 0.832054499
0.767446155 0.977034299 0.658099331
0.489044809 0.170015665 0.176353218
0.076907793 0.311795754 0.479051595
0.282992784 0.671883622 0.115214256
0.383069226 0.131212825 0.376802673
0.641994245 0.255286663 0.996669855
0.8484345 0.687914298 0.064883448
0.638731992 0.590561875 0.357684727
0.93250628 0.304713735 0.200811436
0.302640545 0.638291714 0.590696557
0.038186763 0.751762349 0.381590091
0.12521128 0.335840142 0.982499062
0.905741409 0.929470365 0.851386377
0.490029981 0.13687609 0.694680562
0.340421712 0.826095467 0.902570616
0.703430781 0.237084198 0.534179017
0.838904671 0.649055822 0.580799275
0.104069209 0.361892034 0.706373608
0.652980726 0.52781968 0.837216413

EA131 4.17 4.21 5.76 94 90 120
1.0
4.1674379024 0 0
-2.083318168 3.6605238904 0
-0.0016810623137 -0.45867343316 5.7400975591
6 18
Direct
0.41698392 0.992475567 0.237663608
0.884847102 0.223144536 0.25307939
0.65981669 0.757577395 0.234649469
0.576343237 0.992451193 0.73775198
0.098581011 0.757614803 0.734544341
0.339204164 0.223216122 0.753109334
0.663249581 0.888190452 0.892031125
0.030824187 0.590428366 0.89756266
0.346742895 0.423620248 0.903919008
0.78763907 0.104286302 0.595788054
0.873513836 0.651191919 0.587200741
0.336716022 0.316966147 0.569704028
0.317009212 0.10408342 0.095772986
0.981149843 0.316743933 0.06967866
0.778653723 0.651313305 0.087386816
0.226193791 0.88847082 0.392044339
0.077661766 0.423798178 0.403841385
0.560199878 0.590400536 0.39767204
0.370099601 0.703658249 0.122943008
0.663963649 0.290088207 0.357496299
0.626699656 0.289687354 0.857882193
0.334378477 0.703620828 0.623089379
0.961724229 0.012971458 0.346715547
0.051925552 0.012957651 0.846375871

EA132 4.21 3.34 6.76 97.2 103 107
1.0

4.2114147795 0 0
-0.95616710935 3.1986687937 0
-1.5310801847 -1.3439267479 6.4447993584
6 18
Direct
0.518626201 0.904694075 0.400227643
0.418507374 0.981648138 0.163171818
0.136441419 0.96273131 0.30224019
0.736601198 0.29672394 0.632825173
0.127299951 0.748767725 0.744009508
0.441911012 0.662870623 0.93856127
0.743807023 0.250084915 0.440652062
0.599784296 0.609551745 0.395201073
0.057681912 0.403665752 0.623215813
0.333105572 0.59753535 0.092165704
0.108885775 0.280484106 0.374731173
0.922355313 0.352529027 0.062317331
0.899652609 0.658024064 0.26374013
0.621877102 0.278698906 0.145001947
0.142134582 0.052679919 0.113674815
0.977133417 0.792960676 0.864653486
0.549596259 0.486144853 0.650712434
0.128498307 0.990172799 0.634302407
0.296569719 0.303425143 0.878533289
0.739835907 0.034728038 0.728196314
0.45556002 0.901765931 0.807304969
0.733287438 0.717952771 0.972850761
0.906716183 0.285307611 0.951073174
0.289518681 0.852660862 0.483949631

EA133 4.25 3.34 6.74 87.8 104 112
1.0
4.2504620386 0 0
-1.2609535642 3.0899092578 0
-1.6515304476 -0.38955189795 6.5212828371
6 18
Direct
0.848515425 0.37019466 0.536015652
0.617222398 0.061649181 0.101903805
0.900344119 0.19423252 0.781823061
0.963541575 0.418518345 0.018336742
0.565094981 0.237416483 0.855856225
0.502212319 0.013022785 0.619422283
0.496511004 0.250489187 0.476008965
0.204025981 0.32637432 0.01370204
0.094443975 0.511341956 0.70568573
0.968917156 0.18079504 0.16171936
0.280184406 0.500702527 0.343801067
0.493898312 0.536784938 0.849449981
0.262657833 0.105862889 0.626013116
0.512201199 0.216604038 0.209248023
0.185786313 0.930064184 0.293439539
0.370866345 0.921325517 0.932657125
0.619178679 0.719543055 0.140055439
0.043233547 0.777610407 0.058784012
0.42165825 0.653797254 0.578851831
0.972273586 0.895371914 0.788710004
0.441067301 0.592233014 0.305517474
0.846810677 0.712212355 0.497504208
0.025746659 0.841271888 0.332649774
0.954154923 0.215143504 0.429085209

EA134 4.55 3.21 6.51 89.9 102 111
1.0
4.5464751991 0 0
-1.1310794175 3.0057464282 0
-1.3313308745 -0.49125323756 6.3485057213

```
6 18
Direct
0.577548414 0.244418934 0.27773625
0.244109158 0.57773402 0.611049691
0.810597066 0.988895955 0.394189818
0.477205436 0.322335369 0.727547832
0.910894369 0.911161283 0.944362402
0.143985874 0.655741421 0.060823814
0.773114962 0.842955976 0.553203366
0.440323646 0.176555068 0.886667437
0.378573002 0.27273142 0.356069875
0.045164829 0.60659687 0.689262361
0.675972044 0.293775111 0.649069977
0.614873413 0.390459932 0.118722275
0.510625955 0.732429663 0.744070778
0.543987714 0.834289499 0.261266623
0.009710003 0.960526677 0.316008322
0.210404304 0.167482028 0.594817421
0.844069925 0.399023096 0.410723382
0.281351375 0.723235721 0.451931755
0.947973496 0.057363951 0.785339929
0.877640822 0.501146691 0.92762921
0.711946561 0.939381963 0.022741085
0.177535623 0.065892338 0.077283568
0.106519972 0.509685266 0.219812206
0.343095171 0.627328962 0.982665283
```

--- END ---
